# Structural visualization of key steps in nucleosome reorganization by human FACT

**DOI:** 10.1038/s41598-019-46617-7

**Published:** 2019-07-15

**Authors:** Kouta Mayanagi, Kazumi Saikusa, Naoyuki Miyazaki, Satoko Akashi, Kenji Iwasaki, Yoshifumi Nishimura, Kosuke Morikawa, Yasuo Tsunaka

**Affiliations:** 1 Medical Institute of Bioregulation, Kyushu University, 3-1-1 Maidashi, Higashi-ku, Fukuoka-shi, Fukuoka 812-8582 Japan; 2 Graduate School of Science, Hiroshima University, 1-3-1 Kagamiyama, Higashi-Hiroshima, Hiroshima, 739-8526 Japan; 3 Graduate School of Medical Life Science, Yokohama City University, 1-7-29 Suehiro-cho, Tsurumi-ku, Yokohama 230-0045 Japan; 4 Laboratory of Protein Synthesis and Expression, Institute for Protein Research, Osaka University, 3-2 Yamadaoka, Suita, Osaka 565-0871 Japan; 5 Department of Gene Mechanisms, Graduate School of Biostudies, Kyoto University, Yoshida-konoemachi, Sakyo-ku, Kyoto 606-8501 Japan; 6 Present Address: National Metrology Institute of Japan (NMIJ), National Institute of Advanced Industrial Science and Technology (AIST), 1-1-1 Umezono, Tsukuba, Ibaraki 305-8563 Japan; 7 Present Address: Life Science Center for Survival Dynamics Tsukuba Advanced Research Alliance (TARA), University of Tsukuba, 1-1-1 Tennodai, Tsukuba, Ibaraki 305-8577 Japan

**Keywords:** Chromatin remodelling, Cryoelectron microscopy, Nucleosomes

## Abstract

Facilitates chromatin transcription (FACT) is a histone chaperone, which accomplishes both nucleosome assembly and disassembly. Our combined cryo-electron microscopy (EM) and native mass spectrometry (MS) studies revealed novel key steps of nucleosome reorganization conducted by a Mid domain and its adjacent acidic AID segment of human FACT. We determined three cryo-EM structures of respective octasomes complexed with the Mid-AID and AID regions, and a hexasome alone. We discovered extensive contacts between a FACT region and histones H2A, H2B, and H3, suggesting that FACT is competent to direct functional replacement of a nucleosomal DNA end by its phosphorylated AID segment (pAID). Mutational assays revealed that the aromatic and phosphorylated residues within pAID are essential for octasome binding. The EM structure of the hexasome, generated by the addition of Mid-pAID or pAID, indicated that the dissociation of H2A-H2B dimer causes significant alteration from the canonical path of the nucleosomal DNA.

## Introduction

In eukaryotic genomes, nucleosome is the primary units of chromatin responsible for the restriction of DNA accessibility and the storage of epigenetic information. In nucleosome, a DNA fragment of approximately 145-bp is wrapped around a histone octamer, which consists of one H3-H4 tetramer and two H2A-H2B dimers^1^. The modular nature of nucleosome provides functional complexity through the structural variations, which regulate nuclear architecture and processes, including DNA transcription, replication, and repair. The structural reorganizations at nucleosome or higher order levels are caused by the orchestrated behaviors of ATP-dependent chromatin remodelers and histone chaperones, thereby playing decisive roles in epigenetic regulation^2–4^. Facilitates chromatin transcription (FACT) is a highly conserved histone chaperone, which is essential for both nucleosome assembly and disassembly through the replacement of H2A-H2B dimers^5–7^. For example, *Drosophila* FACT prevents the spreading of heterochromatin through histone replacement at the architectural boundary of chromatin^8^.

**Figure 1:**
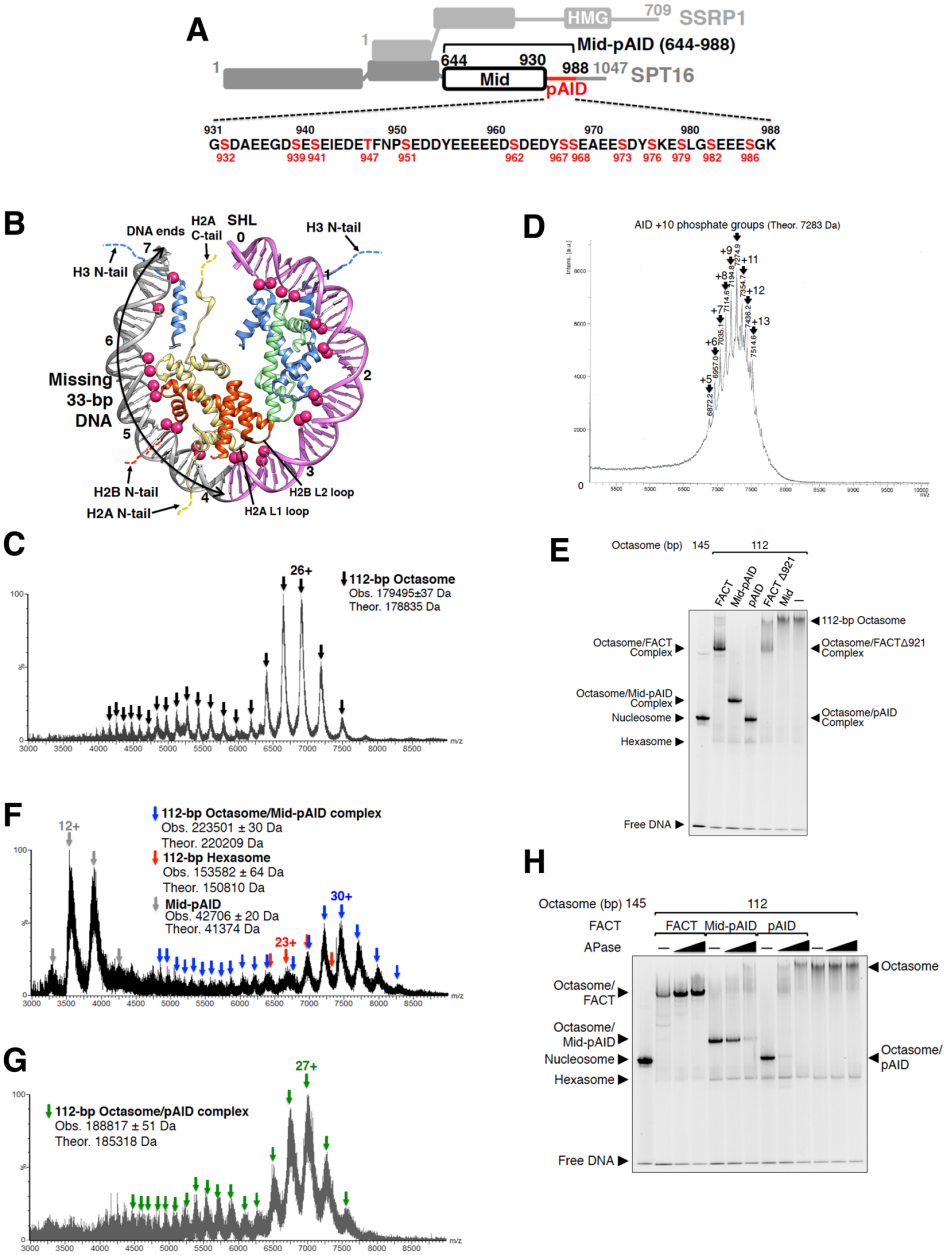
pAID segment of FACT binds to the 112-bp octasome. (**A**) Domain organization of human FACT. The Mid-pAID, Mid, and pAID proteins were used in this structural study. The amino acid sequence of the pAID fragment (residues 931–988) is presented in the lower panel. The potential phosphorylation sites are colored red. (**B**) Approximately one-half of the human nucleosome structure (PDB ID: 2CV5) is shown. Deep pink spheres indicate the DNA phosphate groups forming contacts with histones (H2A, H2B, H3, and H4, colored yellow, red, blue, and green, respectively). The 112-bp DNA is colored orchid. The missing 33-bp DNA is depicted in gray, and is marked by arrows. Dotted lines denote histone tails. Numbers indicate superhelix locations (SHL), which represent the numbers of double turns from the nucleosome dyad (0). (**C**) Native ESI mass spectrum of the 112-bp octasome alone. Arrowheads correspond to multiply charged ions of the 112-bp octasome. The charge state of the mainly observed peak is labeled above the peak. (**D**) MALDI-TOF mass spectrum of the pAID fragment. The mass shift of approximately 80 Da from the theoretical mass of the non-phosphorylated AID (6483 Da) indicates the addition of a phosphoryl group. The observed mass indicated on each peak was consistent with the theoretical mass of multiply phosphorylated AID. The numbers of attached phosphate groups are labeled above the arrowheads. (**E**) EMSAs (electrophoretic mobility shift assays) show the complexes of the 112-bp octasome with the FACT, Mid-pAID, pAID, FACTΔ921, or Mid proteins, detected by SYBR Gold nucleic acid gel stain. The 112-bp octasomes with the FACT proteins showed faster migration than the 112-bp octasome alone, because the negative charge is increased by the complex formation. Experiments were repeated at least three times. (**F**,**G**) Native ESI mass spectra of the 112-bp octasome reconstituted with Mid-pAID (**F**) or pAID (**G**). Blue, red, gray, and green arrowheads show the corresponding peaks for the respective complexes, as indicated. The theoretical masses are calculated from those of the non-phosphorylated proteins. The charge states of the mainly observed peak are labeled above the peak. (**H**) EMSAs show the complexes of the 112-bp octasome with the FACT proteins treated by APase, detected by SYBR Gold nucleic acid gel stain. The complexes between the 112-bp octasome and the FACT proteins were incubated with buffer (−), 0.3 U, or 3 U APase for 2 h at 20 °C. Experiments were repeated at least three times.

The heterodimeric FACT complex consists of the Spt16 and SSRP1 (Pob3 in yeast) subunits, each organized by several conserved domains (Fig. 1A)^9^. The functions of yeast FACT are supported by a separate HMG protein, Nhp6^10,11^. In addition, both C-terminal acidic intrinsically disordered (AID) segments, located in Spt16 and Pob3 (Figs 1A and S1A), are important for the H2A-H2B binding^6^. During transcriptional elongation, FACT is coupled with the ATP-dependent chromatin remodeling enzyme CHD1 (chromodomain-helicase-DNA binding protein 1) and the transcription elongation factor Paf1 (polymerase-associated factor 1), thereby facilitating the passage of RNA polymerase II (RNAPII) through nucleosome^12–15^. Intriguingly, FACT is involved in a broad range of processes, such as DNA transcription, replication, and repair^2,16–19^. In spite of this diverse functional scope, every eukaryote contains only one FACT ortholog, implying that FACT should conduct universal behaviors in terms of nucleosome reorganization.

Our previous studies proposed the mechanism of nucleosome reorganization, involving H2A-H2B eviction by human FACT^20^. First, the AID segment of SPT16 binds to the H2B N-terminal tail (N-tail), detached from the nucleosomal DNA by a double strand break (DSB). Subsequently, the adjacent Mid domain of SPT16 correctly interacts with the H2A docking surface of H3-H4, as revealed from the structure of the Mid-AID protein complexed with an H3-H4 tetramer. Thus, an H2A-H2B dimer is displaced from nucleosome through steric collisions by FACT. Consequently, FACT generates the hexasome by stripping an approximately 30-bp region of the nucleosomal DNA end from histones. On the other hand, the nucleosomal DNA ends can transiently unwrap from histone proteins during a process termed “nucleosome breathing”^21^. In fact, the transcription machinery and ATP-dependent remodelers formed complexes with the partially unwrapped nucleosome^14,22,23^. However, little is known about structures of transient intermediates, which should be leading to further reorganization of nucleosome.

In this report, we present cryo-electron microscopic (EM) snapshots of intermediates and a product, resulting from the action of human FACT. Our study reveals that FACT causes the partial replacement of the nucleosomal DNA end by the densely phosphorylated AID segment (pAID), without alteration of the remaining octasome structure. The combined analyses by cryo-EM and native mass spectrometry (MS) also suggest that the hexasome, lacking an H2A-H2B dimer, is evidently produced through the addition of FACT. In addition, we visualize the significant expansion of the space between two DNA gyres in the hexasome structure. Taken together, these results illustrate how FACT can reorganize nucleosome in the integrated mechanism.

## Results

### Phosphorylated AID segment of FACT binds to the 112-bp octasome

In general, human FACT poorly binds to intact nucleosomes *in vitro*^7,20,24^. However, some perturbations against contacts between histones and the nucleosomal DNA allow FACT to interact with histone core regions covered by DNA^20,24,25^. For example, the introduction of a double-strand break (DSB) into the nucleosomal DNA allows FACT to efficiently form a complex with the DSB nucleosomes^20^. In functional aspects, elongating RNAPII causes transient loss of the histone-DNA contacts through generation of positive torsion against the nucleosomal DNA^26^. In fact, a recent study revealed that *in vivo* transcription produces partially unwrapped DNA as intermediates^27^. Our study aims at visualizing the structures of the complexes formed between the FACT domains and a nucleosome variant with a partially unwrapped DNA. The variant nucleosome, in which a histone octamer is wrapped by DNA shorter than 145-bp, could be regarded as a model for intermediate nucleosomes in the transcriptional process. Such an approach was successfully applied for the mechanistic study of nucleosome reorganization by the Mid-AID region of human FACT (Fig. 1A)^20^. This finding tempted us to examine whether human FACT interacts with a nucleosome variant, which lacks the 33-bp DNA fragment (Fig. 1B). Thus, we reconstituted nucleosome with human histone octamer in the presence of a 112-bp DNA fragment alone by salt-dialysis method. We subsequently examined the histone stoichiometry of the reconstituted nucleosome by native MS. The molecular mass was evaluated to be 179,495 Da (±37), corresponding to the theoretical molecular mass (~179 kDa) for the sum of a histone octamer (two H2A-H2B dimers and one H3-H4 tetramer) and 112-bp DNA (Fig. 1C). This result confirms that the reconstituted nucleosome retains the octameric histones, forming a 112-bp octasome, even though it is missing the 33-bp DNA fragment (Fig. 1B). In addition, the native MS measurement revealed that the octasome exists as a monomer (Fig. 1C). Nevertheless, electrophoretic mobility shift assays (EMSAs) showed a more smeared band of the octasome as compared to that of the 145-bp nucleosome (Fig. S2A). This may arise from the structural polymorphism within the monomeric octasome, such as the heterogeneous positioning of the DNA shorter than 145-bp. In fact, the smeared band became sharper and showed the same migration as that of the 145-bp nucleosome upon the titration of the 33-bp DNA to the 112-bp octasome (Fig. S2A).

The human FACT proteins [Fig. 1A: FACT (SSRP1/SPT16 heterodimer), Mid-pAID, and pAID] were co-expressed in Sf9 insect cells, and purified as reported previously^20^ (Fig. S2B). It should be noted that metazoan FACT is spontaneously phosphorylated in Sf9 insect cells at a comparable level to FACT in the ovary of the female fly^28^. In addition, UV irradiation apparently induced the assembly of the human SPT16/SSRP1/CK2 (casein kinase 2) complex in mammalian cultured cells^16^. Thus, it is quite likely that human FACT is highly phosphorylated when expressed in the cells. In fact, the treatment with calf intestine phosphatase (APase) caused the gel mobility of Mid-pAID to become slower (Fig. S2C), suggesting that APase had removed several of the negatively charged phosphate groups of Mid-pAID. In the crystal structure of the Mid domain^20^, we could not find any phosphorylation sites. Thus, we monitored the extent of the phosphorylation in the AID fragment with MALDI-TOF MS, and found that 5–13 sites were heterogeneously phosphorylated (pAID, Fig. 1D). The pAID segment contains one threonine and twelve serine residues, all of which theoretically could be phosphorylated (Fig. 1A).

EMSAs revealed that human FACT forms stable stoichiometric complexes with the 112-bp octasome (Figs 1E and S2). The same results were observed for the Mid-pAID and pAID proteins (Figs 1E and S3), indicating that pAID alone can interact with the 112-bp octasome. The same stoichiometric complexes were found in native MS measurements for the 112-bp octasomes reconstituted with Mid-pAID (Fig. 1F) and pAID (Fig. 1G). In contrast, the Mid protein alone did not form a complex with the 112-bp octasome (Figs 1E and S3). This result demonstrates that the pAID segment of FACT is essential for the stable complex formation with internal histones through the partial unwrapping of the nucleosomal DNA. In addition, the AID-deleted mutant of FACT, FACTΔ921 [SSRP1/ SPT16 (1–920) complex], reduced the degree of complex formation with the octasome (Figs 1E and S3). This indicates that the pAID segment accomplishes the decisive function of the intact FACT in the 112-bp octasome binding, although the other domains of FACT may weakly interact with the 112 bp octasome. For example, the Pob3-AID segment of the yeast SSRP1 homolog, Pob3, interacts with the H2A-H2B dimer in the same manner as AID of SPT16^6^. Therefore, the phosphorylated SSRP1-AID segment (SSRP1-pAID; Fig. S1A) is likely to play a similar role to the pAID segment of SPT16. As a result, SSRP1-pAID was densely phosphorylated by CK2 (Fig. S1A), and formed the complex with the 112-bp octasome, although the degree of complex formation was lower in comparison with that by pAID of SPT16, as shown in Fig. S1B.

To investigate whether the phosphorylation of pAID affects the octasome binding activity of FACT, we performed EMSAs of the octasome/FACT complexes treated with APase (Fig. 1H). Simultaneously, we estimated the degree of phosphorylation of the FACT proteins after the APase treatment, using ProQ diamond phosphoprotein stain (Fig. S4). The full-length FACT protein maintains the stable complex with the octasome at higher APase concentrations (Fig. 1H). However, the phosphorylation sites of both FACT subunits within the complex are completely protected from the APase action (Fig. S4), thereby retaining the stable complex. In contrast, Mid-pAID and pAID showed obvious reductions of the complex formation with the octasome, in response to the degree of dephosphorylation at each APase concentration (Fig. 1H and S4). These results indicate that the extensive phosphorylation of pAID is crucial for the interaction between FACT and internal histones of the 112-bp octasome.

### Cryo-EM structures of octasomes complexed with Mid-pAID and pAID

**Figure 2:**
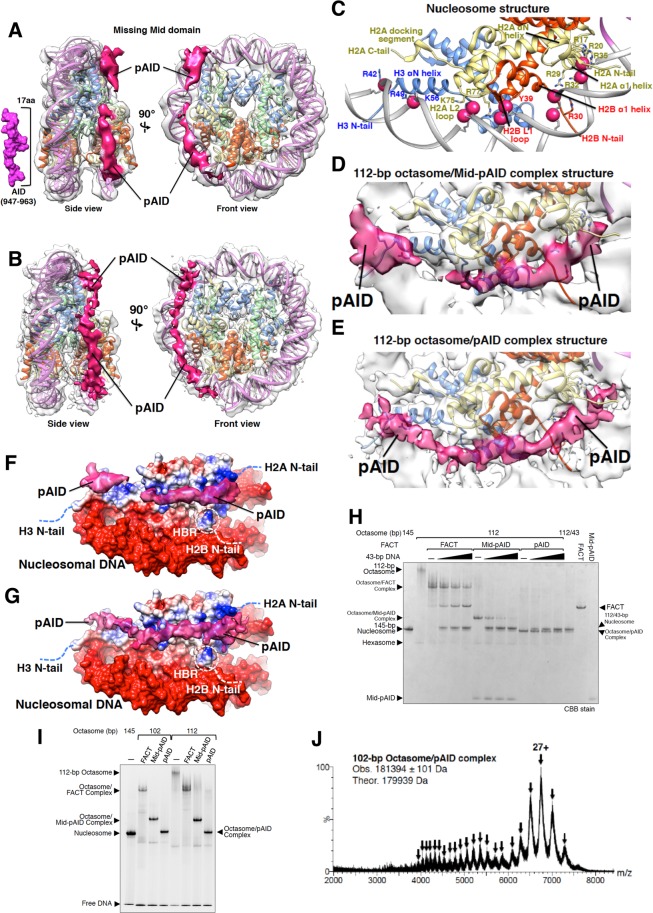
pAID replaces the canonical DNA binding sites with histones within nucleosome. (**A**,**B**) Cryo-EM structures of the 112-bp octasomes complexed with Mid-pAID (**A**) and pAID (**B**), respectively. Two different views of the cryo-EM density map are superimposed onto the nucleosome structure (PDB ID: 2CV5), which lacks the 33-bp DNA (colored as in Fig. 1B). pAID is indicated by a deep pink density. The density of the Mid domain is blurred out during the averaging process in the 3D reconstruction. The surface model corresponding to 17 residues of pAID (947T-963D) is visualized as a scale bar. (**C**–**E**) Detailed views of DNA-histone contacts in nucleosome (**C**) and pAID-histone contacts in the 112-bp octasome/Mid-pAID complex (**D**) and the 112-bp octasome/pAID complex (**E**), colored as in Fig. 1B. The contacts appear to share key residues in histone proteins (R42, R49 and K56 of H3, R30 and Y39 of H2B, and R17, R20, R29, R32, R35, K75, and R77 of H2A). These residues are depicted. (**F**,**G**) Electrostatic surfaces of the pAID binding sites in the 112-bp octasome/Mid-pAID complex (**F**) and the 112-bp octasome/pAID complex (**G**). pAID is indicated by a deep pink density. 112-bp octasomes are shown as electrostatic potential surfaces, colored between −7.0 kT/e (red) and 7.0 kT/e (blue). Dotted lines denote histone tails. White doted circles show the locations of HBR proximate to the H2B N-tail. (**H**) Competitive binding assay between the FACT proteins and the 43-bp DNA performed using the 112-bp octasome, detected by EMSAs. The complexes between the 112-bp octasome and the FACT proteins were titrated with buffer (−), 1.0, 3.3, or 10-fold molar amounts of the 43-bp DNA. The proteins were stained by Coomassie Brilliant Blue (CBB). Experiments were repeated at least three times. (**I**) EMSAs show the complexes of the 102-bp or 112-bp octasomes with the FACT, Mid-pAID, or pAID proteins, detected by SYBR Gold nucleic acid gel stain. Experiments were repeated at least three times. (**J**) Native ESI mass spectrum of the 102-bp octasome reconstituted with pAID. Arrowheads correspond to multiply charged ions of the complex. The charge state of the mainly observed peak is labeled above the peak. The theoretical masses are calculated from those of the non-phosphorylated proteins.

To investigate structural details of the respective complexes with Mid-pAID and pAID, the 112-bp octasomes were reconstituted together with each protein, and were analyzed by cryo-EM (Figs S5A,B and S6). We successfully determined both cryo-EM structures of 112-bp octasomes complexed with Mid-pAID (Figs 2A and S7A) and pAID (Figs 2B and S7B) at overall resolutions of 5.7 Å and 4.5 Å (Table 1 and Figs S5C,D,F,G, S6 and S8A,B,G,H), respectively. The crystal structure of nucleosome^29^ was unambiguously fitted into the density, corresponding to ~112-bp DNA and a histone octamer (Figs S7 and S8D,E). In the higher resolution structure, the density of the histone octamer obviously shows spiral structures for α-helices and possibly some side chains (Fig. S9A). Most notably, we observed obvious extra density in both complex structures (Fig. 2A,B). The density, extending from the H2A N-tail to the H3 N-tail, was quite similar between the two complex structures. On the other hand, we could not observe the density assigned to the Mid domain (Fig. 2A). We presume that this domain would occupy various positions, relative to the major density assigned to the 112-bp octasome and pAID; the averaging process during the 3D reconstruction would have blurred out the density of Mid. Therefore, it is convincing that the extra density in both complexes is assigned to the pAID structures bound to the 112 octasome.

**Table 1:** Cryo-EM Data Collection and Refinement Information.

	112-bp octasome/Mid-pAID complex (Fig. 2A) (EMDB: 6937)	112-bp octasome/pAID complex (Fig. 2B) (EMDB: 9639)	112-bp hexasome (Fig. 4A) (EMDB: 6939)	112-bp octasome/DNA complex (Fig. S14A) (EMDB: 9640)
Microscope	Titan Krios	Titan Krios	Titan Krios	Talos Arctica
Cs corrected	Yes	Yes	Yes	No
Detector	Falcon II	Falcon 3EC	Falcon 3EC	Falcon 3EC
Volta Phase Plate	Yes	No	No	No
Magnification	59,000 (nominal)	59,000 (nominal)	59,000 (nominal)	73,000 (nominal)
Voltage (kV)	300	300	300	200
Electron exposure (e^−^/Å^2^)	36	40	40	40
Defocus range (µm)	−1.00, −1.20, −1.40	−1.75 to −3.50	−1.75 to −3.50	−2.50 to −4.00
Pixel size (Å)	1.1	1.12	1.12	1.4
Initial particle images (no.)	2,62,319	4,23,626	2,29,216	5,48,421
Final particle images (no.)	30,733	1,32,165	42,515	37,441
Map resolution (Å)	5.7	4.5	7.5	7.8
FSC threshold	0.143	0.143	0.143	0.143
Map sharpening *B* factor (Å^2^)	−150	−200	−604	−800

The structural features of the two complexes allow us to interpret that pAID occupies the canonical DNA binding sites of H2A, H2B, and H3 within nucleosome (Fig. 2C–E). pAID makes extensive contacts to traverse basic surfaces of the H2A αN helix, H2A α1 helix, H2B N-tail, H2B α1 helix, H2B L1 loop, H2A L2 loop, and H3 αN helix (Fig. 2D–G). Thus, extensive phosphorylation sites of pAID are likely to be involved in interactions with key residues of internal histones (R42, R49 and K56 of H3, R30 and Y39 of H2B, and R17, R20, R29, R32, R35, K75, and R77 of H2A, Fig. 2C–E), which are shared for binding to ten phosphate groups of the nucleosomal DNA. In fact, the higher resolution structure appears to exhibit possible interactions between pAID and key histone residues (Fig. S9B–D). To understand the property of the pAID interaction with the octasome, we carried out a competitive binding assay between the FACT proteins and a 43-bp DNA, using the 112-bp octasome (Fig. 2H). Upon the addition of DNA, all of the FACT proteins were dissociated from the complexes with the 112-bp octasome. Simultaneously, the 112/43-bp DSB nucleosome bands appeared (Fig. 2H). This result demonstrates that the pAID segment within the complex is replaceable with DNA.

On the other hand, the core structures of the histone octamer in the 112-bp octasome/Mid-pAID and octasome/pAID complexes are mostly unaltered in comparison with the intact nucleosome (Figs 1B, 2A,B and S8D,E), although the side chains that interact with pAID are blurred on the density map at the current resolution. This suggests that the replacement of DNA by pAID minimally affects the octameric histone core structure in nucleosome. The H3 αN helix, which is essential for retaining the nucleosomal DNA ends within nucleosome, is clearly visualized in both complexes (Fig. 2D,E). Similarly, the H2A αN helix is ordered (Fig. 2D,E), although it is disordered in the solution structure of an H2A-H2B dimer without DNA^30^. These findings indicate that these helices are stabilized by interactions with pAID in place of DNA. Notably, in the complex structures, pAID contacts the N-terminal proximal segment of H2B (Fig. 2D,E), which corresponds to a highly basic and conserved H2B repression region (HBR, residues 27–34 in H2B). This is in agreement with the previous report that HBR is essential for FACT binding to H2A–H2B^31^. HBR is sandwiched between the two gyres of the nucleosomal DNA and locks the histone octamer into the canonical position on the nucleosomal DNA^32^. Therefore, in the 112-bp octasome alone, the unlocking of HBR by the lack of DNA may cause the structural polymorphism of the octasome (Fig. S2A). This interpretation is consistent with the previous report that reduced contacts of histones with DNA facilitate DNA sliding^33^. However, the location of histone octamer remains the same in the 112-bp octasome/Mid-pAID and octasome/pAID complexes (Figs 1B, 2A,B and S8D,E), presumably because HBR is held between pAID and DNA (Fig. 2F,G). Taken together, the pAID segment appears to fix the octameric histone structure at the correct position.

In a previous study, DNA unwrapping over ~40-bp length led to the spontaneous conversion from nucleosome to hexasome^21^. In fact, the 43-bp DNA unwrapping completely eliminated the histone-DNA contacts on H2A-H2B (Fig. S10). To confirm whether pAID prevents the spontaneous histone displacement by the missing 43-bp DNA, we reconstituted the 102-bp octasome with pAID, Mid-pAID, and FACT. It was difficult to obtain the 102-bp octasome alone, because insoluble aggregates were formed during the salt dialysis. In contrast, the 102-bp octasomes successfully formed stable complexes with the FACT proteins in EMSAs (Fig. 2I). We subsequently examined the histone stoichiometry of the 102-bp octasome/pAID complex by native MS (Fig. 2J). The molecular mass was evaluated to be 181,394 Da (±101), indicating that the complex retains the octameric histones and pAID (the theoretical molecular mass: ~180 kDa).

Furthermore, we investigated the behaviors of the disordered histone tails in the 112-bp octasome/pAID complex formation, using the respective deletion mutants of the N-terminal disordered region (N-tail) of histones H3 (1–35) and H2B (1–25) (Fig. S11). The deletion of the H3 N-tail (H3_ΔN-tail) weakly affected the formation of both complexes with 33-bp DNA and pAID, as compared to the intact H3 (Fig. S11). Therefore, the H3 N-tail appears to have a role in the formation of both complexes with DNA and pAID. However, the deletion of the H2B N-tail (H2B_ΔN-tail) hardly formed the complex with pAID (Fig. S11). This highlights the importance of the H2B N-tail for the complex formation with pAID, in good agreement with our previous model, where FACT initially binds to the H2B N-tail through pAID^20^.

### Aromatic and phosphorylated residues within pAID are essential for octasome binding

**Figure 3:**
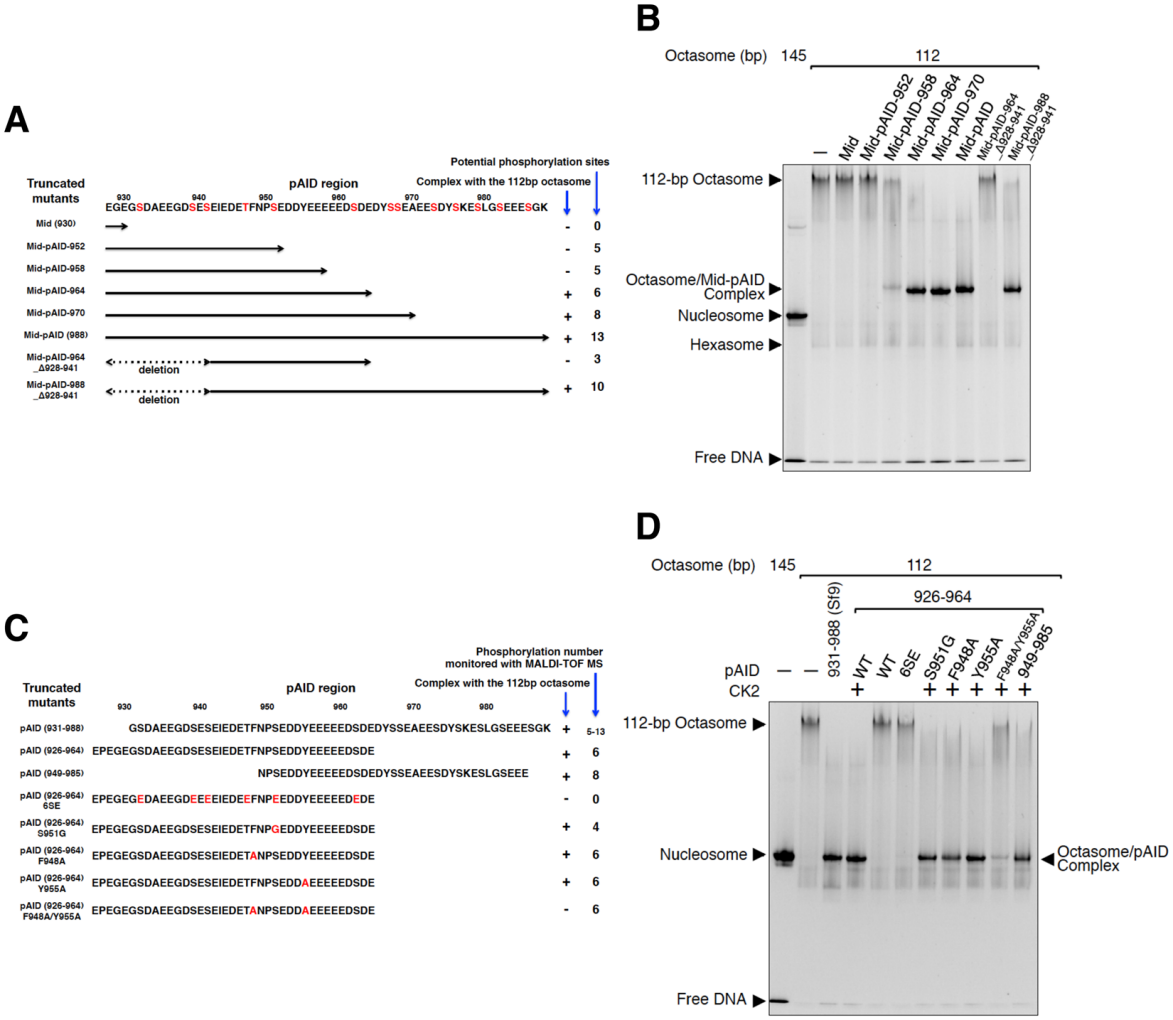
Systematic analysis of the complex formation of the 112-bp octasome with the mutants of Mid-pAID and pAID. (**A**) Schematic drawing of the truncated mutants of Mid-pAID. These mutants were used in (**B**). The amino acid sequence of SPT16 (residues 928–988) is presented. The potential phosphorylation sites are colored red. (**B**) EMSAs show the complexes of the 112-bp octasome with the truncated mutants of Mid-pAID, detected by SYBR Gold nucleic acid gel stain. Experiments were repeated at least three times. (**C**) Schematic drawing of the pAID mutants. These mutants were used in (**D**). The amino acid sequence of SPT16 (residues 926–988) is presented. The mutated residues are colored red. (**D**) EMSAs show the complexes of the 112-bp octasome with the mutants of pAID, detected by SYBR Gold nucleic acid gel stain. Experiments were repeated at least three.

To restrict the essential motifs in the pAID sequence, we performed systematic octasome binding assays, using truncated mutants of Mid-pAID and pAID (Fig. 3). The pAID density is roughly divided into two sub-regions, located around the H3 N-tail and along the H2A-H2B dimer (Fig. 2A,B). Notably, the latter density has a similar length to the model of 17 residues of AID (947–963) (Fig. 2A). When combining these sub-regions, the density appears to match the atomic model of approximately 34 residues of pAID. This interpretation is consistent with the results that the pAID lengths (>34 residues) of Mid-pAID are critical for the complex formation (Fig. 3A,B). In addition, each of the N-terminal (39 residues: 926–964) and C-terminal (37 residues: 949–985) pAID segments independently formed stable complexes with the 112-bp octasome (Fig. 3C,D). These results suggest that pAID may have two histone-binding elements composed of approximately 17 residues, which are ascribed to the central segment (949–964) and the N-terminal or C-terminal segments (926–948 or 965–985), respectively. Unfortunately, it is difficult to conclusively adjust the two binding elements to the pAID density, as the local resolutions range from 5 to 9 Å in the cryo-EM map (Fig. S8B).

To identify the specific residues responsible for histone contacts, we performed additional EMSAs, using the residue mutants of the truncated pAID segment (926–964) (Fig. 3C,D). The 6SE mutant, in which all six of the phosphorylation sites (S932, S939, S941, T947, S951, and S962) are replaced by acidic residues (glutamate), showed no complex formation with the 112-bp octasome (Fig. 3D). This highlights the notion that the binding requires the phosphorylated serine/threonine residues, which may mimic the phosphate backbones of DNA. Notably, the S951G mutant, which expands the spacing between the phosphorylated residues, formed a stable complex (Fig. 3D), implying that the slight reduction of the phosphorylation minimally affects the interaction. On the other hand, the previous structure indicated that a single aromatic residue in pAID of yeast SPT16 is important for the H2A-H2B binding^6^. Thus, we investigated the effects of two aromatic residues within the truncated pAID. Whereas the single mutants (F948A and Y955A) formed complexes, the F948A/Y955A mutant abolished the complex formation (Fig. 3D). This result suggests that the interaction conforms to a double-anchor model for APLF (Aprataxin and Polynucleotide kinase Like Factor) binding to H2A-H2B^34^. Collectively, we conclude that the complex formation depends upon the specific binding, involving the two aromatic residues and the extensive phosphorylation.

### FACT causes partial conversion from octasome to hexasome

**Figure 4:**
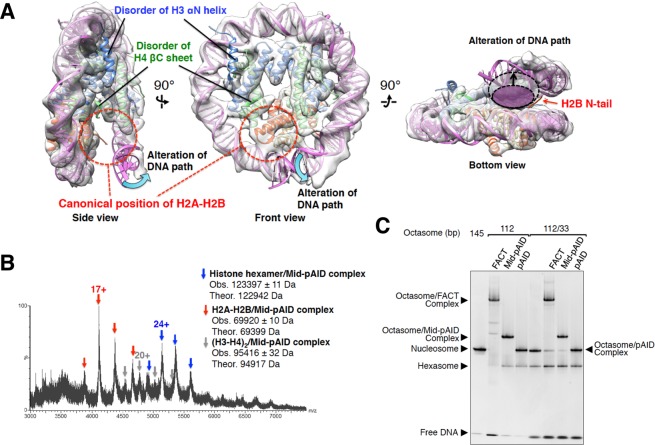
FACT causes partial conversion from octasome to hexasome. (**A**) Cryo-EM structure of the 112-bp hexasome. Three different views of the cryo-EM density map are superimposed onto the nucleosome structure (PDB ID: 2CV5), which lacks the 33-bp DNA and one H2A-H2B dimer. Red dotted circles show the canonical position of H2A-H2B in nucleosome (Side and Front views). Indicated structures of the H3 αN helix and the H4 βC sheet are disordered in the 112-bp hexasome map (Side and Front views). The cyan curved arrows show the significant alteration of the nucleosomal DNA path (Side and Front views). The dashed ellipsoids, shadowed in white and magenta, represent the space between two DNA gyres within the hexasome and octasome, respectively (Bottom view). The black arrows indicate the expanding distance between the hexasome and octasome (Bottom view). (**B**) Native ESI mass spectrum of the histone octamer mixed with Mid-pAID. Blue, red, and gray arrowheads show the corresponding peaks for the respective complexes, as indicated. The theoretical masses are calculated from those of the non-phosphorylated proteins. The charge states of the mainly observed peak are labeled above the peak. (**C**) EMSAs show hexasome formation upon binding of the FACT proteins to the 33/112-bp DSB nucleosome, detected by SYBR Gold nucleic acid gel stain. They are compared with the hexasome products in the 112-bp octasome reconstituted with the FACT proteins. Experiments were repeated at least three times.

In the cryo-EM analyses of both 112-bp octasomes reconstituted with Mid-pAID and pAID, we found extra particles in addition to the above complexes (Figs S5A,B and S6). The reconstructed images of the particles revealed the hexasome structure, which is missing an H2A-H2B dimer and the FACT proteins (Figs 4A, S5E and S6). The density of the resulting hexasome at an overall resolution of 7.5 Å (Table 1 and Figs S5H and S8C,I) is well fitted by ~106-bp DNA and the protein secondary structures of an H3-H4 tetramer and an H2A-H2B dimer (Figs 4A and S8F). Our cryo-EM map visualized the hexasome structure at a higher resolution than those reported previously^21^ (Fig. S12), and thus clearly shows α-helical structures of histones and DNA grooves. The hexasome structure is missing an H3 αN helix, whose structure is retained in nucleosome by interactions with the H2A docking segment and the nucleosomal DNA end (Fig. 4A). In the crystal structure of the overlapping dinucleosome, the H3 αN helix in the hexasome region, lacking the H2A-H2B dimer, is partially ordered^35^, presumably because the helix interacts with the DNA backbone of the adjacent octasome. An H4 C-terminal segment is also disordered which forms a β-sheet with the N-terminal proximal moiety of the H2A docking segment in the intact nucleosome (Fig. 4A). Notably, the H2A-H2B dissociation leads to the loss of the DNA contacts with the H2A L1 and H2B L2 loops (Fig. 1B), thereby altering the DNA path through a release from bending constraints on the canonical DNA path within nucleosome (Fig. 4A). Furthermore, the DNA path alteration results in the significant expansion of the space between a DNA end and its nearby DNA gyre in the hexasome structure (Fig. 4A). These structural rearrangements of histones and DNA in the hexasome are likely to be caused by the dissociation of the H2A-H2B dimer from the octasome and the shorter length of the nucleosomal DNA.

To clarify whether the hexasome structure results from the addition of the FACT proteins or a destabilizing effect under the cryo-EM conditions, we performed the cryo-EM analysis for the 112-bp octasome alone (Figs S13 and S14). As a result, we found the structures of irregular octasomes (Fig. S14A), which would be artificially produced by the cryo-EM procedure with the denaturing tendency. However, we could not find any obvious hexasome structures (Fig. S13E). In contrast, the hexasome structures were observed with a high frequency in the cryo-EM analyses of the 112-bp octasome with the FACT proteins (Fig. S6). Therefore, it seems highly possible that the observed hexasome structure is formed by the addition of the FACT proteins, although we could not completely exclude the possibility that the hexasome is artificially produced by a destabilizing effect under the cryo-EM conditions.

Our previous report indicated that the Mid domain of FACT binds to the H2A docking surface of an H3-H4 tetramer in nucleosome, thereby displacing an H2A-H2B dimer from nucleosome through steric collisions^20^. Thus, the addition of Mid-pAID may partially cause the conversion from octasome to hexasome. In good agreement with this scenario, the native MS measurements for the 112-bp octasome reconstituted with Mid-pAID found the slight existence of the 112-bp hexasome components (Fig. 1F). In contrast, the molecular mass of the hexasome was hardly detected in the native mass spectrum for the 112-bp octasome reconstituted with pAID alone (Figs 1G and S15)^36^. The hexasome structures were observed with a higher frequency in the cryo-EM analyses of the 112-bp octasome with Mid-pAID [Fig. S6: 34.9% of dataset 1 (Classes 1, 3, 6, and 10) and 53.8% of dataset 2 (Classes III, V, and VII)] than those with pAID [Fig. S6: 27.0% of dataset 1 (Classes 5 and 6) and 19.3% of dataset 2 (Classes 1 and 8)]. However, in the EMSAs, the addition of Mid-pAID to the 112-bp octasome exhibited a quite low efficiency of hexasome formation, similar to that of pAID alone (Fig. 1E). In addition, the hexasome formation hardly changed throughout the titrations of FACT, Mid-pAID, and pAID in the EMSAs (Fig. S3B,C). This implies that the interaction between FACT and the 112-bp octasome is firmly stabilized through holding the H2B N-tail between pAID and DNA, as observed by cryo-EM (Fig. 2F,G). The cryo-EM and native MS procedures may tend to perturb the complexes with the FACT proteins in EMSAs.

According to our previous structure of the Mid-AID/(H3-H4)_2_ complex^20^, Mid additionally causes steric hindrance with the nucleosomal DNA at a position ~10-bp distant from the dyad axis in the nucleosome structure (Fig. S16). Thus, the resultant local peeling of the nucleosomal DNA near the dyad axis may be required for the efficient invasion of Mid into nucleosome. In fact, a histone octamer mixed with Mid-pAID in the absence of DNA was observed to produce a histone hexamer complexed with Mid-pAID, as revealed by the native MS analysis (Fig. 4B). In addition, the hexasome formation by Mid-pAID was more efficient in the 33/112-bp DSB nucleosome than in the 112-bp octasome reconstituted with Mid-pAID (Fig. 4C). Thus, it is likely that the behavior of Mid-pAID at the DSB site leads to the higher efficiency of hexasome production, as described in the following section.

## Discussion

**Figure 5:**
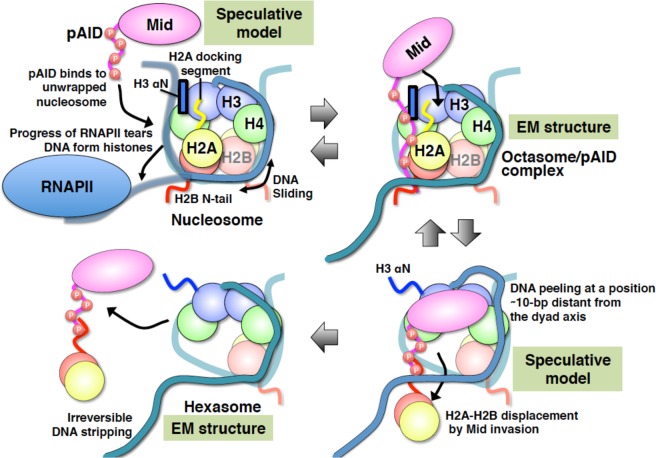
Summary of the integrated mechanism for nucleosome reorganization by Mid-pAID of FACT. Histone proteins and DNA in nucleosomes are indicated by different colors as follows: H2B (red), H2A (yellow), H3 (blue), H4 (green), and DNA (light sea green). Both Mid and pAID are colored magenta. RNAPII is colored dodger blue. The blue box represents the αN helix of H3. Disordered regions of proteins are indicated by colored strings. The red open circles labeled with P indicate the phosphorylation sites.

Our biochemical and structural findings allow us to envisage mechanistic views in nucleosome reorganization by human FACT (Fig. 5). At the first step, the pAID segment of FACT binds to the unwrapped nucleosome, which is subjected to actions of transcription machinery and ATP-dependent remodelers. In place of the peeled-off DNA, pAID compensates for the loss of histone-DNA contacts without alteration of the internal histone structure, as revealed in the EM structure (Fig. 2A,B). This replacement is a trigger for the Mid domain of FACT to subsequently invade into nucleosome, although the Mid invasion appears to require other perturbation. Eventually, the Mid invasion causes the hexasome formation and the irreversible stripping of 30-bp DNA.

The transient DNA unwrapping is a dynamically executed process. However, it is not until missing of 33-bp DNA that FACT can bind to nucleosome (Fig. 1E). This result suggests that FACT is not responsible for replacing DNA in the transiently unwrapping nucleosome. Presumably, the unwrapped nucleosome could exist in transcriptional complexes, consisting of various molecular machineries, such as the ATP-dependent remodeler CHD1^14^. In fact, the structural snapshots of nucleosomal transcription revealed that the progress of RNAPII tears DNA from histones^23^. Therefore, FACT can bind to such unwrapped nucleosome (Fig. 5 upper left).

Whereas the DNA unwrapping over ~40-bp leads to spontaneous hexasome formation^21^, the native MS measurement showed that the 102-bp octasome/pAID complex retains octameric histones (Fig. 2J). Accordingly, FACT appears to prevent the spontaneous removal of histones, the aggregations on its own, and the nucleosome sliding by such unwrapping DNA (Fig. 5 upper right). In other words, this implies that FACT constitutively maintains the histone-based epigenetic memory throughout transcription and replication. In fact, FACT prevented the H2A-H2B displacement during *in vitro* transcription^37^. In a recent study, *in vivo* depletion of FACT caused a striking increase in 90-bp particles, arising from the loss of histones, at the +1 nucleosome position of transcription^27^. This indicates that the deletion of FACT induces substantial unwrapping of the nucleosomal DNA and concomitant loss of histones in transcription *in vivo*. Similarly, FACT prevented accumulation of histone variant H2A.Z and nucleosome loss in transcribed regions of yeast^38^. In replication machinery, the loss of FACT also severely inhibited chromatin replication in the fully reconstituted system, and the addition of FACT to the replication system was sufficient for complete replication^39^. These reports are consistent with our mechanistic view.

Subsequently, FACT may enable Mid to invade nucleosome, thereby displacing H2A-H2B. The Mid binding to nucleosome appears to require structural alterations, such as the local peeling of the nucleosomal DNA at a position ~10-bp distant from the nucleosome dyad (Fig. 5 lower right and S13)^20^. Our previous report revealed that the invasion of Mid-pAID into the DSB nucleosome occurs in concert with the detachment of a 33-bp DNA segment from histone^20^. Notably, the DSB site is located near the H2B N-tail, which is buried by the two gyres of DNA and fixes the histone position on DNA^32^. Therefore, it is likely that the detachment of the H2B N-tail from DNA upon the Mid-pAID binding induces the structural perturbation of the DSB nucleosome^20^. In agreement, SPT16 displaced H2A-H2B dimers to disrupt the outer nucleosomal wrap when DNA was strained^40^. Presumably, such DNA strain may require the Mid invasion to nucleosome.

FACT may be involved in further disruption of nucleosome beyond the hexasome (Fig. 5 lower left), as implied from the EM structure of the 112-bp hexasome with the remarkable expansion of the space, where the DNA end is released from the H2A-H2B binding constraints within nucleosome (Fig. 4A). It may also be possible that the further expansion of the space causes the detachment of the nearby H2B N-tail from DNA (Fig. 4A), thereby resulting in the structural and dynamic polymorphism of the hexasome. In this context, we envisage that the inherent function of FACT could be coupled with the action of ATP-dependent chromatin remodelers. In fact, human FACT efficiently assists the chromatin remodeler, RSC, to destabilize nucleosome structures, as revealed by the high accessibility to restriction enzymes^41^. In addition, the chromatin remodeler, Fun30/SMARCAD1, collaborates with FACT to induce nucleosome disassembly at transcribing regions during RNAPII transcription^42^ and in the mismatch repair system using *Xenopus* egg extracts^43^.

Intriguingly, Mid appears to play important roles in nucleosome assembly. In fact, a recent study indicated that the K692A/R693A mutations in the Mid domain impaired the deposition of newly synthesized histones H3-H4 in yeast^44^. Similarly, extracts from cells with the Mid mutations displayed a defect of nucleosome assembly in the DNA supercoiling assay. The conserved residues in human (K674 and R675) are mapped onto a highly conserved and basic surface of the Mid structure (Fig. S16)^20^. The docking model between Mid and nucleosome indicates that the broad basic surface of Mid contacts the nucleosomal DNA around the central region (Fig. S16). Therefore, the mutations are likely to prevent FACT from depositing the nascent H3-H4 onto the replicating DNA.

In conclusion, our structural study has made an important advance for understanding the mechanisms of nucleosome assembly and disassembly by FACT. In addition, our structural snapshots provide unprecedented insights into integrity and versatility in epigenetic regulatory processes involving FACT. In this connection, it should be noted that eukaryotic cells universally contain only one ortholog of FACT. Therefore, our mechanistic views could be generally applied to nucleosome reorganization in various nuclear processes, whose progressions are impeded by nucleosomes. Therefore, FACT appears to be the most central player to dictate nucleosome reorganization processes, which proceed correctly through the integrated mechanism proposed by us.

## Methods

### Protein expression and purification

Recombinant human histone proteins were produced in *E. coli* and purified as reported previously^29^. Baculovirus-driven expressions of the human FACT complex (co-expression of N-terminal His-tagged SPT16 and non-tagged SSRP1), of FACTΔ921 [co-expression of N-terminal His-tagged SPT16 (1–920) and non-tagged SSRP1], and of the mutants (N-terminal His-tagged hMid-AID, the truncated mutants, and hMid) in Sf9 insect cells were carried out as reported previously^20^. The FACT proteins were purified according to previously published protocols^20^. All DNA segments are based on the 601 nucleosome positioning sequence^45^. DNA fragments of 33-bp, 43-bp, 102-bp, 112-bp, and 145-bp were constructed and purified as described previously^20^. A 33/112-bp DSB nucleosome, 145-bp nucleosomes (intact, H3_ΔN-tail, and H2B_Δ-Ntail), 112-bp octasomes (intact, H3_ΔN-tail, and H2B_Δ-Ntail), a 112-bp octasome/Mid-pAID complex, a 112-bp octasome/pAID complex, a 112-bp octasome/FACT complex, a 102-bp octasome/Mid-pAID complex, a 102-bp octasome/pAID complex, and a 102-bp octasome/FACT complex were reconstituted from histones, DNAs, and the FACT proteins by the salt dialysis method, and then purified as described previously^29^. The truncated pAID (926–964), pAID (949–985), and SSRP1-pAID (434–514) proteins were expressed in *Escherichia coli* strain BL21 (DE3) containing the expression plasmid pColdI (pAID) with or without pRSFduet (CK2), as reported previously^46^. Cells were suspended in buffer containing 150 mM NaCl, 20 mM Tris-HCl, pH 8.5, and 1 mM DTT, and lysed by sonication. The pAID proteins were partially purified on a HisTrap column (GE Healthcare) and then the His tags were removed by Factor Xa protease (NEB). The pAID proteins were applied to a HiTrap Q anion exchange column (GE Healthcare). Fractions containing the pAID proteins were further purified on a Superdex200 GF column (GE Healthcare). The extent of phosphorylation was determined for the pAID segments by mass measurement using MALDI-TOF MS (Autoflex, Bruker, Billerica, MA).

### EMSAs

The 112-bp octasomes (1.5 pmol) were mixed with the 33-bp DNA or various FACT constructs (0.75, 1.5, or 3 pmol) in a reaction buffer containing 150 mM NaCl, 20 mM Tris-HCl, pH 8.5, and 1 mM DTT, and incubated for 15 min at 30 °C. The products in the 102-bp or 112-bp octasomes reconstituted with each FACT protein (1.5 pmol) were also incubated in the buffer for 15 min at 30 °C. In the APase assay, Mid-pAID and the 112-bp octasomes reconstituted with the FACT proteins (1.5 pmol) were incubated with buffer (−), 0.3 U, or 3 U APase (Calf intestine phosphatase, Takara-Bio) for 2 h at 20 °C. In the competition assay, the 112-bp octasomes reconstituted with the FACT proteins (1.5 pmol) were titrated with buffer (−), 1.0, 3.3, or 10-fold molar amounts of the 43-bp DNA, and incubated for 15 min at 30 °C. The 33/112-bp DSB nucleosomes (1.5 pmol) were mixed with the FACT proteins (FACT full, 3 pmol; others, 10 pmol), and incubated for 15 min at 30 °C. These were compared with the products in the 112-bp octasomes reconstituted with each FACT protein (1.5 pmol). The samples were electrophoresed at 4 °C on a 7.5% native-PAGE in 1 × Tris-Glycine buffer, and then were visualized by SYBR Gold nucleic acid gel stain or Coomassie Brilliant Blue (CBB) stain.

### Native ESI mass spectrometry

The nano ESI mass spectra and ion mobility arrival times were acquired with a Tri-wave SYNAPT G2 HDMS mass spectrometer (Waters, Milford, MA) with a nanoESI source, as described previously^35,36,47^. For native ESI mass spectrometry, the samples containing the 112-bp octasome were dialyzed against 4 mM Tris-HCl, pH 7.6, and then diluted with concentrated ammonium acetate, resulting in a 2 µM analyte solution in 0.5–1.5 mM Tris-HCl and 200 mM ammonium acetate. The solution of histone octamer mixed with Mid-pAID was dialyzed against 500 mM ammonium acetate and then diluted with 500 mM ammonium acetate, resulting in a 2 µM analyte solution. The samples were deposited in a nanospray tip prepared in-house^35,36,47^, and was placed in the nanoESI source. To observe the ions of the sample, the backing pressure was increased up to ~5 mbar by closing the SpeediValve. The temperature of the ion source was set at 70 °C. The sample cone and collision voltages were set at 30–50 V and 15 or 20 V, respectively. The mass spectra were acquired for m/z 2,000–14,000, calibrated with (CsI)nCs^+^ ions. To analyze the arrival times by ion mobility mass spectrometer, the ion mobility wave velocity was set at 800 m/s and the wave height was set at 40 V. The MassLynx version 4.1 software (Waters) was used for data processing and peak integration.

### Cryo-EM sample preparation

Holey carbon (Quantifoil R1.2/1.3 Au 200) and holey gold grids were used for frozen hydrated specimens. Holey gold grids (estimated gold thickness ~10 nm) were obtained by gold sputtering of Quantifoil grids (R1.2/1.3 Au 200), using an MC1000 sputtering device (Hitachi High Tech Inc.) equipped with a quartz crystal microbalance (QCM) thickness monitor. Grids were glow discharged for 1 min by an HDT-400 hydrophilic treatment device (JEOL) before usage. Two microliters of sample solutions were applied on holey grids for rapid freezing. Holey gold grids were used for 112-bp octasome reconstituted with Mid-pAID (hereinafter referred to as “octasome/Mid-pAID”). Holey carbon grids were used for 112-bp octasome reconstituted with pAID (hereinafter referred to as “octasome/pAID”) and 112-bp octasome alone (hereinafter referred to as “octasome”). Rapid freezing was performed using an EM-GP freezing robot (Leica).

### Cryo-EM data collection

All of the samples were first examined for optimization of the sample preparation conditions, using an FEI Polara electron microscope operated at 200 kV accelerating voltage. Images were taken using either an UltraScan 2k CCD camera with a Gatan GIF Tridiem energy filter (GATAN) or an UltraScan 4k CCD camera (GATAN) (Fig. S13A,B).

Electron microscopic image datasets for structure analyses of octasome/Mid-pAID and octasome/pAID were collected on a Titan Krios electron microscope (Thermo Fisher Scientific), equipped with a spherical aberration (Cs) corrector (CEOS GmbH), under 300 kV accelerating voltage, using the EPU software (Thermo Fisher Scientific) for fully automated data acquisition. The samples of octasome/Mid-pAID were examined by using a Falcon II direct electron detector (Thermo Fisher Scientific). Images were recorded in the dose-fractionation mode (2 sec exposure/32 fractions), at a nominal magnification of 59,000x, corresponding to 1.10/pixel on the specimen (Fig. S5A). A total electron dose of 36 electrons/Å^2^ was used for each image recording. A Volta phase plate (VPP) was used to enhance the contrast of particle images. The nominal defocus was in the range of −1.0 µm ~−1.4 µm. The samples of octasome/pAID were examined by using a Falcon 3EC direct electron detector (Thermo Fisher Scientific). Images were recorded in the electron counting mode (Fig. S5B), at a nominal magnification of 59,000x, corresponding to 1.12 Å/pixel on the specimen, with a total electron dose of 40 electrons/Å^2^ and 62 sec total exposure time. The defocus range of the data was −1.75 µm~−3.50 µm. Intermediate frames (total 2,410 frames) were recorded every 0.60 sec, giving an accumulated dose of 40 electrons/Å^2^ and a total of 100 fractions per image.

Electron microscopic images of octasome were collected on a Talos Arctica electron microscope (Thermo Fisher Scientific) using a Falcon 3EC direct electron detector (Thermo Fisher Scientific). Images were recorded in the electron counting mode (Fig. S13C), at a nominal magnification of 73,000x, corresponding to 1.4 Å/pixel on the specimen, with a total electron dose of 40 electrons/Å^2^ and 73 sec total exposure time. The defocus range of the data was −2.5 µm~−4.0 µm. Intermediate frames (total 2,836 frames) were recorded every 0.51 sec, giving an accumulated dose of 40 electrons/Å^2^ and a total of 140 fractions per image.

### Cryo-EM image processing

Movie frames for octasome/Mid-pAID, octasome/pAID, and octasome were aligned to correct the dose-induced motions of the specimens and dose-weighted using MotionCor2^48^. The Contrast Transfer Function (CTF) was determined for each image using the CTFFIND4 program^49^. About one thousand particle images were picked manually from the images using the RELION manual picking tools.

The initial small dataset was subjected to reference-free classification in RELION^50,51^. Selected “good” class average images were used as references to automatically pick particles, using Gautomatch (http://www.mrc-lmb. cam.ac.uk/kzhang/Gautomatch/) or the autopicking tool of RELION. The output coordinates of the picked particles were used for particle extraction program of RELION. The extracted images were subjected to reference-free 2D classification (Class2D, RELION) to remove bad images. The initial 3D volumes were obtained by initial volume reconstructing tools in EMAN2^52^ or RELION.

From the images of octasome/Mid-pAID, 262,319 particles were extracted and subjected to two rounds of 2D classification/particle selection procedure (Fig. S6A, dataset 1). In total, 122,955 particles were subjected to the 3D classification procedure in RELION (Class3D), assuming ten classes. Particles classified to the best (class #8, 30,733 particles) and second best (class #6, 13,880 particles) 3D classes, corresponding to an octasome and a hexasome, respectively, were further used for refining (Refine3D in RELION) each of the two class 3D maps. The refined map of the octasome (class #8) was sharpened by applying a negative B-factor (−150) and corrected for the MTF of the Falcon II detector. The final map of the octasome (class #8) indicated the structure of the 112-bp octasome/Mid-pAID complex, as shown in Figs 2A and S6A. The Gold standard FSC of the final map was 5.7 Å (Fig. S5F).

The class 3D map of class #6 was the hexasome, which corresponds to a product of nucleosome generated by the action of FACT domains. As the resolution of the refined map was not high enough, we increased the number of movies (1,459) and particles (284,455) (Fig. S6A, dataset 2). After two rounds of screening particles by selecting good classes obtained by reference-free 2D class averaging in RELION, 163,898 particle images were used for 3D classification. Class #II (octasome) and class #III (hexasome) exhibited relatively high resolution structures. A total of 40,058 particles, classified as the hexasome structure (class #III), were used for 3D auto-refine (RELION), followed by B-factor (−200) sharpening, as well as MTF correction. The resolution estimated by the Gold standard FSC was 7.7 Å (class #III). The refined map of the octasome (class #II, 34,456 particles) exhibited a structure very similar to the above described 5.7 Å map; however, its resolution remained at the lower value of 7.0 Å.

The structure analysis of octasome/pAID was performed using two datasets. First, 229,216 particle images were extracted, and bad particles were removed using the 2D classification result (Fig. S6B left, dataset 1). In total, 228,121 particles from the images of octasome/pAID were used for 3D classification (eight classes, Fig. S6B left). Class #5 and class #8 exhibited essentially the same structure of the hexasome and the octasome, respectively, as obtained by the sample of octasome/Mid-pAID. A total of 42,515 particles, classified to the hexasome (class #5), were further refined by the same procedure and corrected for the MTF of the Falcon 3EC detector. Automated B-factor sharpening^53^ was applied to the map (B-factor = −604). The final map of the hexasome (class #5) indicated the structure of the 112-bp hexasome, as shown in Figs 4A and S6B left. The Gold standard FSC resolution of the final map was 7.5 Å (Fig. S5H).

A total of 96,459 particles, classified as the octasome (class #8), were subjected to another round of 3D classification, assuming four classes (Fig. S6B left). This procedure has been applied to check whether this group could be classified to different structure, and to exclude bad images from the datasets assigned to this group. While classes #I, #II, and #III exhibited high resolution structures similar to each other, class #IV showed a rather low resolution map, and thus the particles belonging to this class were removed. Since we could not detect significant differences between the three classes with higher resolutions, particle images, belonging to classes #I (29,915 particles), #II (30,125 particles), and #III (25,585 particles), were merged (total 85,625 particles) and used for refinement. The MTF corrected and B-factor sharpened map indicated the structure of the 112-bp octasome/pAID complex, and the resolution was 5.6 Å (Fig. S6B left).

Second, particle images were added to improve the resolution of the octasome/pAID map (Fig. S6B right, dataset 2). From the newly dataset 2, 199,410 particles were extracted and subjected to 2D classification/particle selection procedure. A total of 162,273 particles were classified in eight 3D classes. The class #7 map exhibited a high-resolution 3D map of octasome-pAID and particles assigned to this class (66,560 particles) were joined with the particles classified as the octasome in the dataset 1 (85,625 particles), after Movie refinement and Particle polishing procedure in RELION. The merged 152,185 particles were subjected to 3D classification assuming four classes, to check the variability and to remove bad particles. As shown in Fig. S6B right, while class II, III, and IV exhibited excellent 3D map with essentially the same structure, the 3D map of Class I did not reach high resolution, thus particles belonging to this class were removed from further analysis. After 3D refinement, MTF correction, and B-factor sharpening procedure (B-factor = −200), the final map indicated the structure of the 112-bp octasome/pAID complex (Figs 2B and S6B right). The Gold standard FSC resolution of the final map was 4.5 Å (Fig. S5G).

Images of octasome (the 112-bp octasome alone), recorded by a CCD camera, mostly showed aggregates (Fig. S13A), oligomers (Fig. S13B), and dissociated DNA strands (Fig. S13B). The extracted images were subjected to reference-free 2D class averaging in RELION. As a result, particles with the mono-nucleosome size were partly observed (Fig. S13B,D). Particle images with sizes similar to mono-nucleosome, recorded by a Falcon 3EC direct electron detector (Fig. S13C), were extracted (548,421 particles) and subjected to 3 rounds of 2D classification/particle selection procedure to remove bad particle images such as those of nucleosome aggregations, and images affected by the DNA in the background. The selected 57,514 particles were subjected to 3D classification, assuming three classes (Fig. S13E). Among the three classes, only class 1 exhibited a high resolution and interpretable structure. After 3D refinement and MTF correction, and B-factor sharpening (B = −800), the final map indicated the structure of the 112-bp octasome complexed with an extra DNA (Fig. S14A). The Gold standard FSC resolution of the final map was 7.8 Å (Fig. S13G).

Visualization of the 3D maps and fitting of crystal structures into the obtained maps were performed using Chimera^54^. Electrostatic surface potentials were calculated using APBS^55^. The extended loop structure of pAID (931G-988K) was built using the PDB utility Server (http://spin.niddk.nih.gov/bax/nmrserver/pdbutil/ext.html) at National Institute of Diabetes and Digestive and Kidney Diseases. This structure was used as the reference for calculating 20 atomic models of pAID by MODELLER integrated in Chimera^56^. The most extended structure was selected and the surface model corresponding to 17 residues of AID (947T-963D) is depicted as a scale bar in Fig. 2A.

## Supplementary information

Supplementary Figure S1-S16

## Data Availability

The accession numbers for the cryo-EM reconstructions of the 112-bp octasome/Mid-pAID complex, the 112-bp octasome/pAID complex, the 112-bp hexasome complex, and the 112-bp octasome/DNA complex reported in this paper are EMDB: 6937 (Fig. 2A), EMDB: 9639 (Fig. 2B), EMDB: 6939 (Fig. 4A), and EMDB: 9640 (Fig. S14A), respectively.
