# Supplementary material for: Structural visualization of key steps in nucleosome reorganization by human FACT
Source: Sci Rep. 2019 Jul 15;9:10183. doi: 10.1038/s41598-019-46617-7 (PMC6629675; doi:10.1038/s41598-019-46617-7)
Supplement: Supplementary file 1 — Supplementary Figure S1-S16 [file 41598_2019_46617_MOESM1_ESM.pdf]

Supplementary Material

Title

Structural visualization of key steps in nucleosome reorganization by human FACT

Author names and affiliations

Kouta Mayanagi,[1,*] Kazumi Saikusa,[2,3,#] Naoyuki Miyazaki,[4] Satoko Akashi,[3] Kenji Iwasaki,[4,§] Yoshifumi Nishimura,[3] Kosuke Morikawa,[5,*] and Yasuo Tsunaka[3,*]

[1]Medical Institute of Bioregulation, Kyushu University, 3-1-1 Maidashi, Higashi-ku, Fukuoka-shi, Fukuoka 812-8582, Japan.

[2]Graduate School of Science, Hiroshima University, 1-3-1 Kagamiyama, Higashi-Hiroshima, Hiroshima 739-8526, Japan.

[3]Graduate School of Medical Life Science, Yokohama City University, 1-7-29 Suehiro-cho, Tsurumi-ku, Yokohama 230-0045, Japan.

[4]Laboratory of Protein Synthesis and Expression, Institute for Protein Research, Osaka University, 3-2 Yamadaoka, Suita, Osaka 565-0871, Japan.

[5]Department of Gene Mechanisms, Graduate School of Biostudies, Kyoto University, Yoshida-konoemachi, Sakyo-ku, Kyoto 606-8501, Japan.

*Corresponding Author

maya@bioreg.kyushu-u.ac.jp (K.Ma.), morikawa.kosuke.2x@kyoto-u.ac.jp (K.Mo.), tsunaka@yokohama-cu.ac.jp (Y.T.)

Lead Contact (Y.T.)

Graduate School of Medical Life Science, Yokohama city University,

1-7-29 Suehiro-cho, Tsurumi-ku, Yokohama 230-0045, Japan.

TEL: +81-45-508-7381

FAX: +81-45-508-7360

Mail: tsunaka@yokohama-cu.ac.jp

[#]Present address: National Metrology Institute of Japan (NMIJ), National Institute of Advanced Industrial Science and Technology (AIST), 1-1-1 Umezono, Tsukuba, Ibaraki 305-8563, Japan

[§]Present address: Life Science Center for Survival Dynamics Tsukuba Advanced Research Alliance (TARA), University of Tsukuba, 1-1-1 Tennodai, Tsukuba, Ibaraki 305-8577, Japan

Figure S1

pAID segment of SSRP1 binds to the 112-bp octasome.

(A) Domain organization of human FACT. The SSRP1-pAID protein was used in this study. The amino acid sequence of the SSRP1-pAID segment (residues 434–514) is presented in the upper panel. The potential phosphorylation sites are colored red.

(B) EMSAs show the complexes of the 112-bp octasome with the SSRP1-pAID or SPT16-pAID proteins, detected by SYBR Gold nucleic acid gel stain. Experiments were repeated at least three times.

Figure S1

A

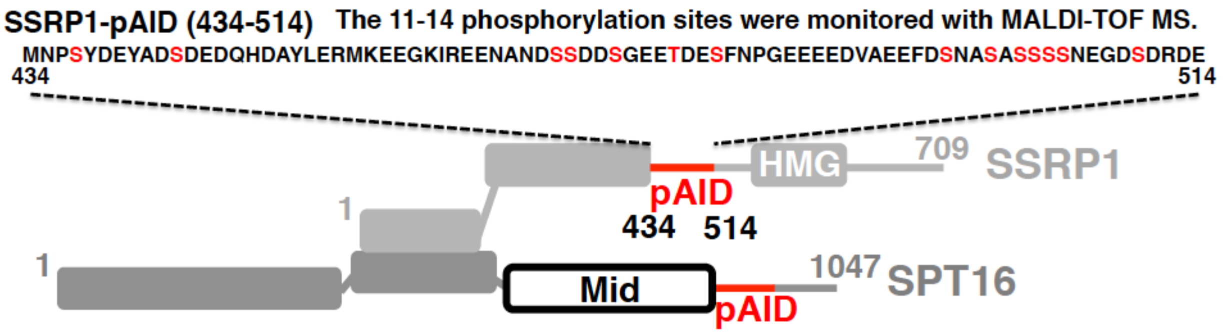
SSRP1-pAID (434-514) The 11-14 phosphorylation sites were monitored with MALDI-TOF MS.
MNPSYDEYADSDEDQHDAYLERMKEEGKIREENANDSSDDSGEETDESFNPGEEEEDVAEEFDSNASASSSSNEGDSDRDE
434
514
HMG
709
SSRP1
pAID
434 514
1
1
Mid
1047
SPT16
pAID

B

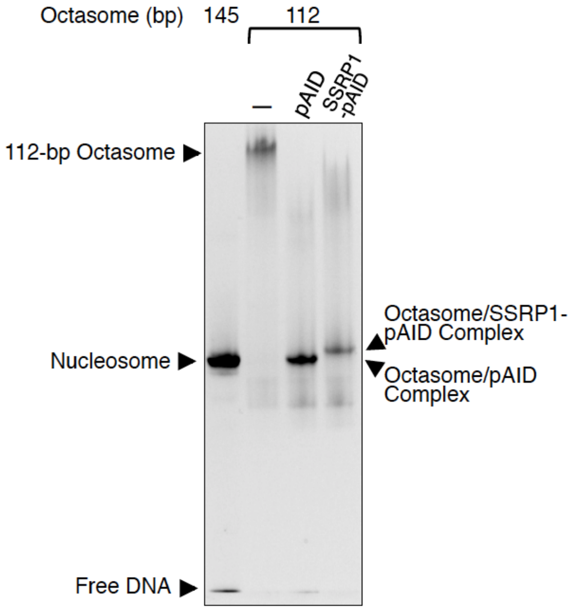
Octasome (bp) 145 112
—
pAID
SSRP1-pAID
112-bp Octasome
Nucleosome
Free DNA
Octasome/SSRP1-pAID Complex
Octasome/pAID Complex

Figure S2

Sample conditions.

(A) EMSAs show the complexes of the 112-bp octasome (1.5 pmol) with the 33-bp DNA (0.75, 1.5, 3 pmol), detected by SYBR Gold nucleic acid gel stain. The migration behavior in EMSAs is defined by the molecular charge and size. The 112-bp octasome has an obviously lower negative charge in comparison with that of the 145-bp nucleosome. In addition, its molecular weight (~180 kDa) is slightly different from that of nucleosome (~200 kDa). In this case, the effect of charge is relatively dominant. As a result, the band of the 112-bp octasome shows much slower migration than that of the 145-bp nucleosome. Experiments were repeated at least three times.

(B) CBB staining of the purified FACT proteins (1.5 pmol) after 15% SDS-PAGE. Molecular mass is depicted in kDa. The pAID proteins were not stained by CBB, because of the highly negative charge cluster. Thus, the pAID proteins were confirmed by mass measurements using MALDI-TOF MS (Figures 1D and 3C)

(C) EMSAs show that APase causes the gel mobility of human Mid-pAID to become slower, detected by CBB stain. The Mid-pAID proteins (1.5 pmol) were incubated with buffer (-), 0.3 U, 0.6 U, 1.2 U, or 3 U APase for 2h at 20 °C. Experiments were repeated at least three times.

Figure S2

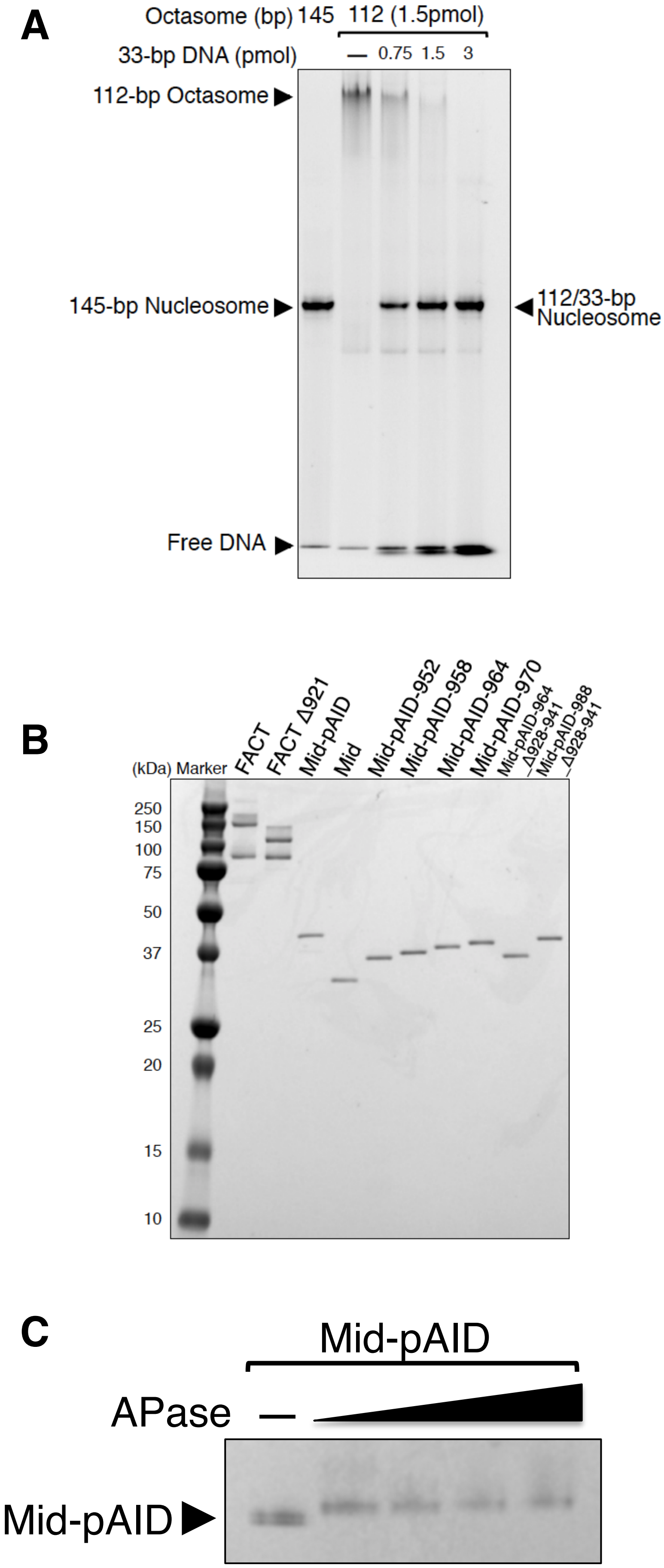
A
Octasome (bp) 145 112 (1.5pmol)
33-bp DNA (pmol) — 0.75 1.5 3
112-bp Octasome
145-bp Nucleosome
112/33-bp Nucleosome
Free DNA
B
(kDa) Marker
FACT
FACT Δ921
Mid-pAID
Mid
Mid-pAID-952
Mid-pAID-958
Mid-pAID-964
Mid-pAID-970
Mid-pAID-964_Δ928-941
Mid-pAID-988_Δ928-941
250
150
100
75
50
37
25
20
15
10
C
Mid-pAID
APase —
Mid-pAID

Figure S3

Titration of the FACT proteins to the 112-bp octasome.

(A) CBB staining of a 15 % SDS-PAGE gel, showing how much protein was present in the lanes in Figure 1E. The pAID protein was not stained by CBB, because of the highly negative charge cluster. Molecular mass is depicted in kDa.

(B, C) SYBR Gold stained EMSAs (B) and the CBB stained duplicate (C) for the titration of the FACT proteins (0.75, 1.5, 3 pmol) to the 112-bp octasome (1.5 pmol). The FACT, Mid-pAID, and pAID proteins formed the complex with the octasome at a 1:1 stoichiometry. In contrast, the AID-deleted FACT protein (FACTΔ921) clearly exhibited the remaining band from the free octasome even at the 2:1 stoichiometry. The Mid protein hardly interacted with the octasome at any stoichiometry. The free pAID protein was not stained by CBB, because of the highly negative charge cluster. The free Mid protein was not applied in the EMSA, because of its positively charged property. Experiments were repeated at least three times.

Figure S3

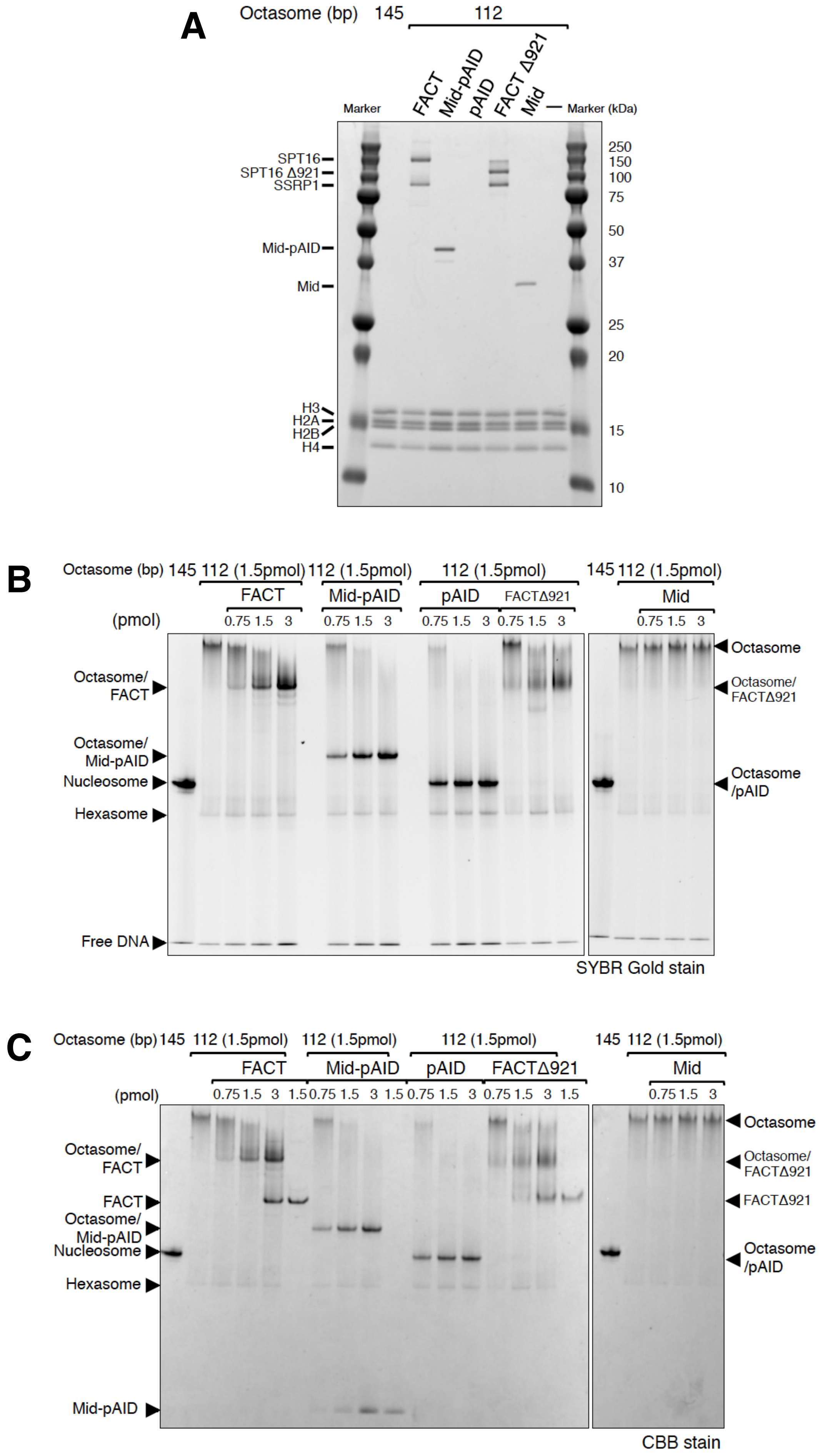
A
Octasome (bp) 145 112
Marker
FACT
Mid-pAID
pAID
FACT Δ921
Mid
—
Marker (kDa)
SPT16
SPT16 Δ921
SSRP1
Mid-pAID
Mid
H3
H2A
H2B
H4
250
150
100
75
50
37
25
20
15
10
B
Octasome (bp) 145 112 (1.5pmol) 112 (1.5pmol) 112 (1.5pmol) 145 112 (1.5pmol)
FACT Mid-pAID pAID FACTΔ921 Mid
(pmol) 0.75 1.5 3 0.75 1.5 3 0.75 1.5 3 0.75 1.5 3 0.75 1.5 3
Octasome/ FACT
Octasome/ Mid-pAID
Nucleosome
Hexasome
Free DNA
Octasome
Octasome/ FACTΔ921
Octasome /pAID
SYBR Gold stain
C
Octasome (bp) 145 112 (1.5pmol) 112 (1.5pmol) 112 (1.5pmol) 145 112 (1.5pmol)
FACT Mid-pAID pAID FACTΔ921 Mid
(pmol) 0.75 1.5 3 1.5 0.75 1.5 3 1.5 0.75 1.5 3 0.75 1.5 3 1.5 0.75 1.5 3
Octasome/ FACT
FACT
Octasome/ Mid-pAID
Nucleosome
Hexasome
Mid-pAID
Octasome
Octasome/ FACTΔ921
FACTΔ921
Octasome /pAID
CBB stain

Figure S4

Analysis of the phosphorylation levels of the FACT proteins, using ProQ diamond stain. The degrees of phosphorylation of the FACT proteins with the 112-bp octasome (7 pmol), in a 15% SDS-PAGE gel after the APase treatment (1.4 or 14 U), were detected by ProQ diamond phosphoprotein stain (left). The CBB stained duplicate is shown on the right. As a result, pAID was obviously dephosphorylated at lower APase concentrations. Mid-pAID was clearly dephosphorylated at higher APase concentrations. In contrast, the FACT proteins were hardly dephosphorylated at any APase concentration. Therefore, it is possible that the phosphorylation sites within pAID are protected from APase action by the other domain of FACT. The free pAID protein was not stained by CBB, because of the highly negative charge cluster. Experiments were repeated at least three times.

Figure S4

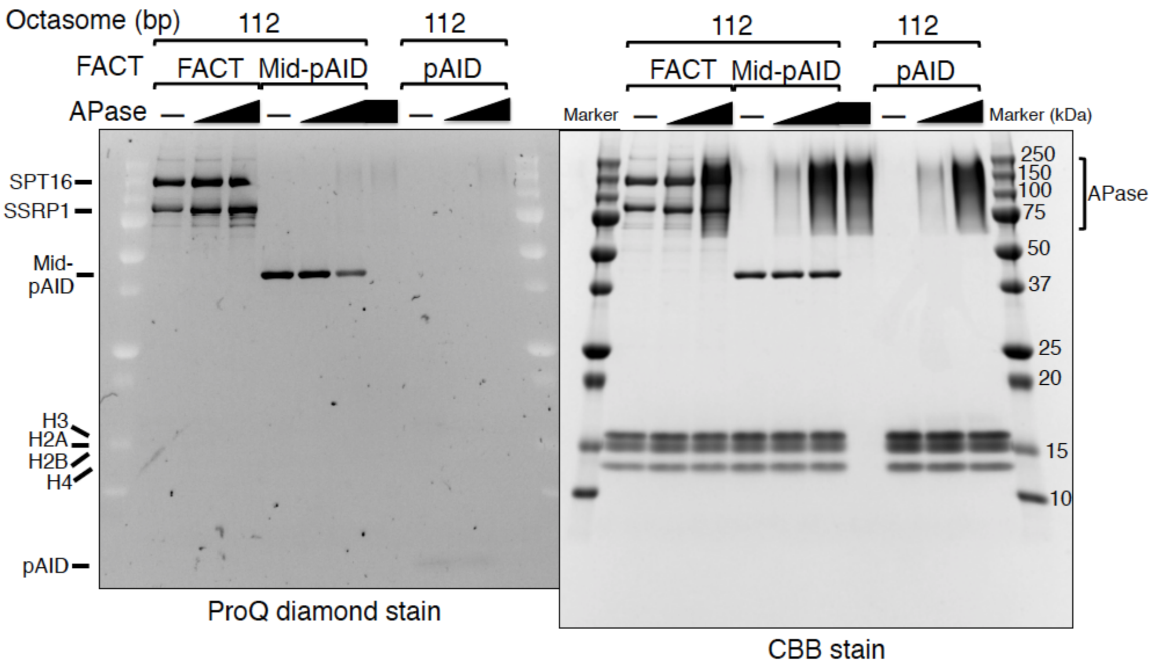

Octasome (bp)
112
112
112
112
FACT
FACT
Mid-pAID
pAID
FACT
Mid-pAID
pAID
APase
Marker
Marker (kDa)
SPT16
SSRP1
Mid-pAID
H3
H2A
H2B
H4
pAID
250
150
100
75
50
37
25
20
15
10
APase
ProQ diamond stain
CBB stain

Figure S5

Cryo-EM analysis.

(A) Representative cryo-electron micrograph of the 112-bp octasome reconstituted with Mid-pAID, recorded by a Falcon II direct electron detector, using VPP.

(B) Representative cryo-electron micrograph of the 112-bp octasome reconstituted with pAID, recorded by a Falcon 3EC direct electron detector using the electron counting (EC) mode, without VPP.

(C, D, E) Comparison between representative 2D class averages (left) and projections from the 3D model (right) of the 112-bp octasome/Mid-pAID complex (C), the 112-bp octasome/pAID complex (D), and the 112-bp hexasome (E).

(F, G, H) Fourier shell correction (FSC) curves for the EM density maps of the 112-bp octasome/Mid-pAID complex (F), the 112-bp octasome/pAID complex (G), and the 112-bp hexasome (H). Resolutions are given for the FSC 0.143 criteria.

Figure S5

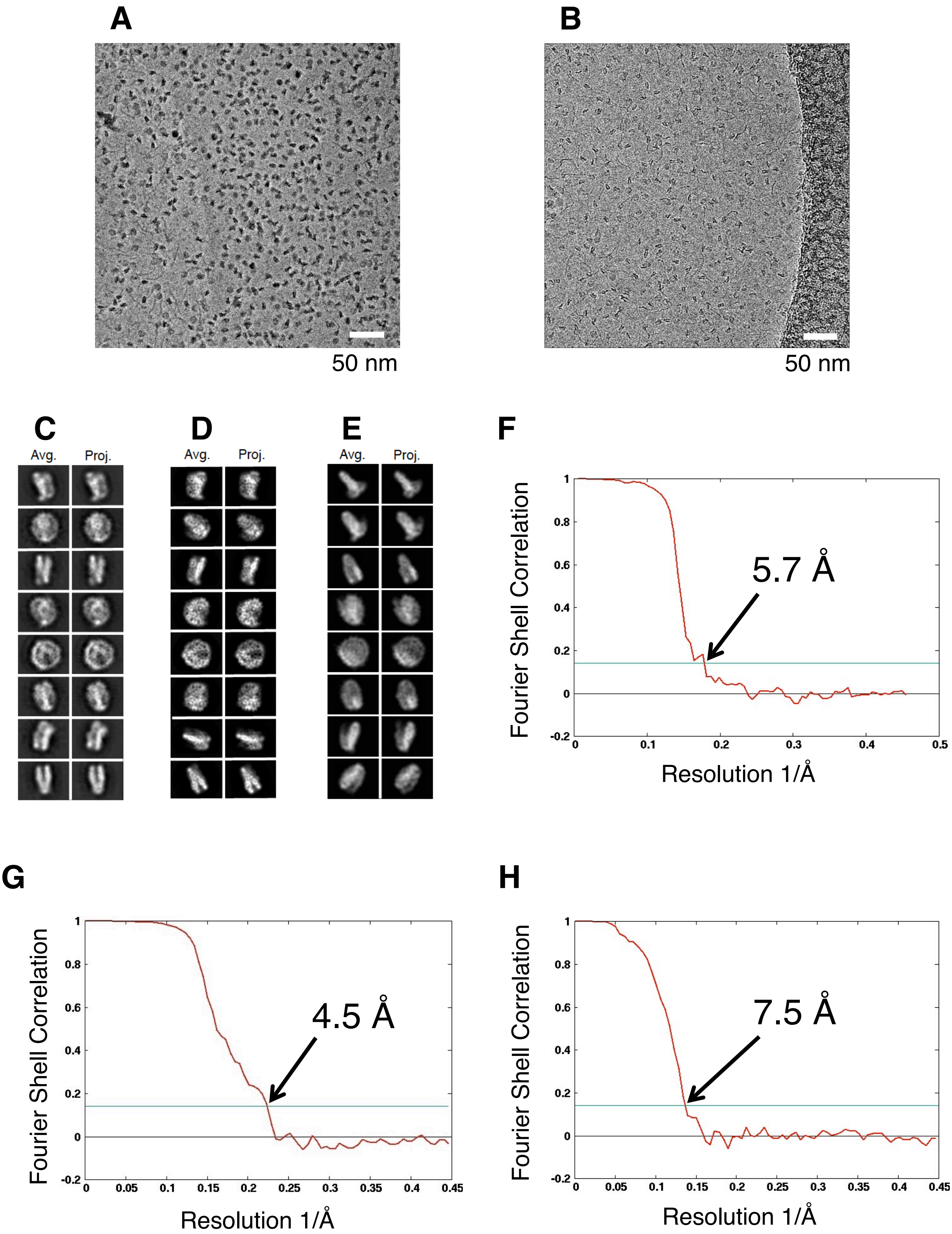
A
50 nm
B
50 nm
C
Avg.
Proj.
D
Avg.
Proj.
E
Avg.
Proj.
F
Fourier Shell Correlation
Resolution 1/Å
5.7 Å
G
Fourier Shell Correlation
Resolution 1/Å
4.5 Å
H
Fourier Shell Correlation
Resolution 1/Å
7.5 Å

Figure S6

Flow charts of cryo-EM data processing.

(A) 3D classification and refinement procedures for the 112-bp octasome reconstituted with Mid-pAID. The details are described in Methods. EM structures viewed from front and back sides are shown in their classification scheme.

(B) 3D classification and refinement procedures for the 112-bp octasome reconstituted with pAID. The details are described in Methods. EM structures viewed from front and back sides are shown in their classification scheme.

Figure S6

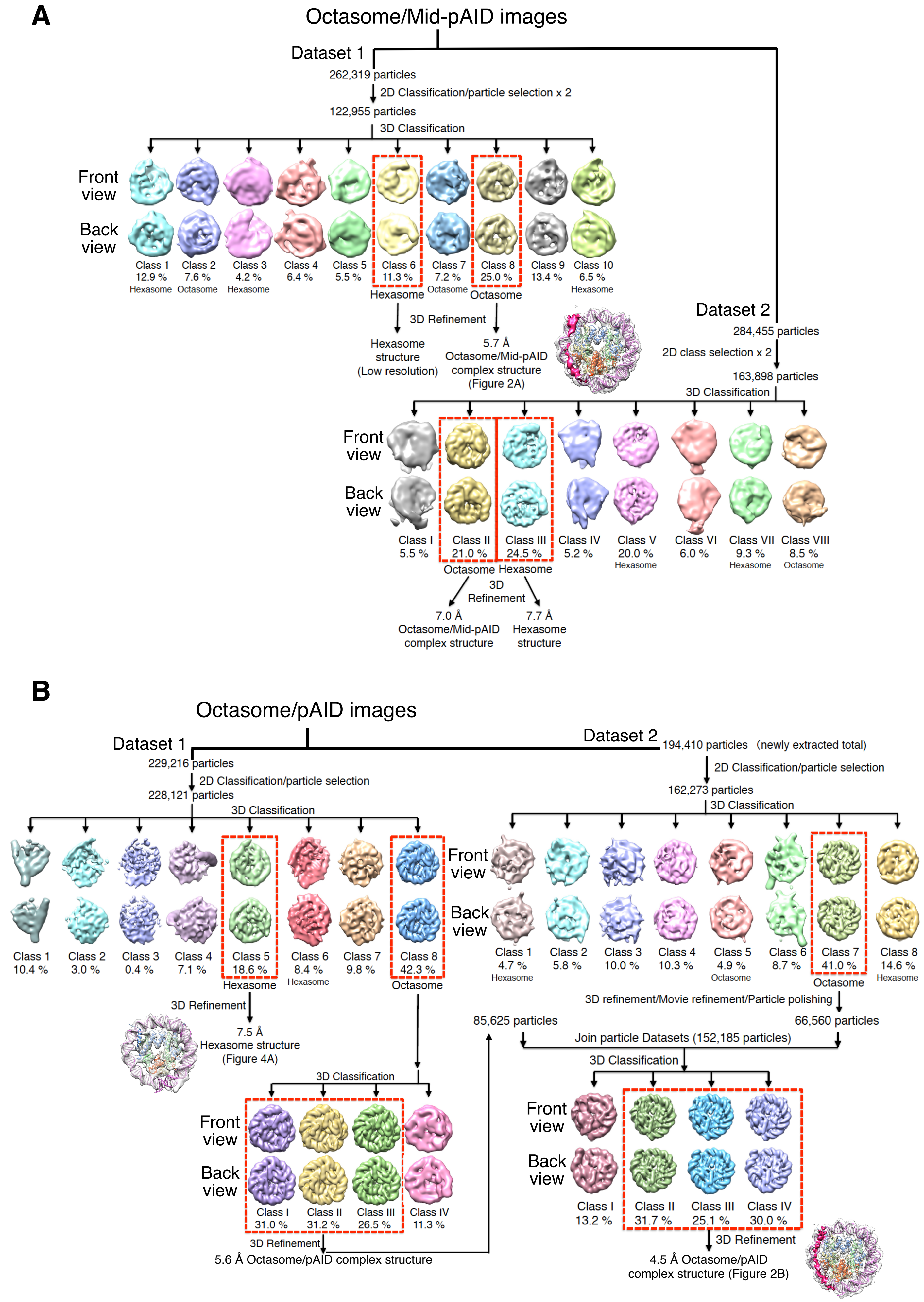

A
Octasome/Mid-pAID images
Dataset 1
262,319 particles
2D Classification/particle selection x 2
122,955 particles
3D Classification
Front view
Back view
Class 1 12.9 % Hexasome
Class 2 7.6 % Octasome
Class 3 4.2 % Hexasome
Class 4 6.4 %
Class 5 5.5 %
Class 6 11.3 % Hexasome
Class 7 7.2 % Octasome
Class 8 25.0 % Octasome
Class 9 13.4 %
Class 10 6.5 % Hexasome
3D Refinement
Hexasome structure (Low resolution)
5.7 Å Octasome/Mid-pAID complex structure (Figure 2A)
Dataset 2
284,455 particles
2D class selection x 2
163,898 particles
3D Classification
Front view
Back view
Class I 5.5 %
Class II 21.0 % Octasome
Class III 24.5 % Hexasome
Class IV 5.2 %
Class V 20.0 % Hexasome
Class VI 6.0 %
Class VII 9.3 % Hexasome
Class VIII 8.5 % Octasome
3D Refinement
7.0 Å Octasome/Mid-pAID complex structure
7.7 Å Hexasome structure
B
Octasome/pAID images
Dataset 1
229,216 particles
2D Classification/particle selection
228,121 particles
3D Classification
Class 1 10.4 %
Class 2 3.0 %
Class 3 0.4 %
Class 4 7.1 %
Class 5 18.6 % Hexasome
Class 6 8.4 % Hexasome
Class 7 9.8 %
Class 8 42.3 % Octasome
Front view
Back view
3D Refinement
7.5 Å Hexasome structure (Figure 4A)
3D Classification
Front view
Back view
Class I 31.0 %
Class II 31.2 %
Class III 26.5 %
Class IV 11.3 %
3D Refinement
5.6 Å Octasome/pAID complex structure
Dataset 2
194,410 particles (newly extracted total)
2D Classification/particle selection
162,273 particles
3D Classification
Class 1 4.7 % Hexasome
Class 2 5.8 %
Class 3 10.0 %
Class 4 10.3 %
Class 5 4.9 % Octasome
Class 6 8.7 %
Class 7 41.0 % Octasome
Class 8 14.6 % Hexasome
3D refinement/Movie refinement/Particle polishing
85,625 particles
66,560 particles
Join particle Datasets (152,185 particles)
3D Classification
Front view
Back view
Class I 13.2 %
Class II 31.7 %
Class III 25.1 %
Class IV 30.0 %
3D Refinement
4.5 Å Octasome/pAID complex structure (Figure 2B)

Figure S7

Rear views of the cryo-EM structures.

(A, B) Cryo-EM structures of the 112-bp octasomes complexed with Mid-pAID (A) and pAID (B), respectively. Rear views of the cryo-EM density map are superimposed onto the nucleosome structure (PDB ID: 2CV5), which lacks the 33-bp DNA (colored as in Figure 1B).

Figure S7

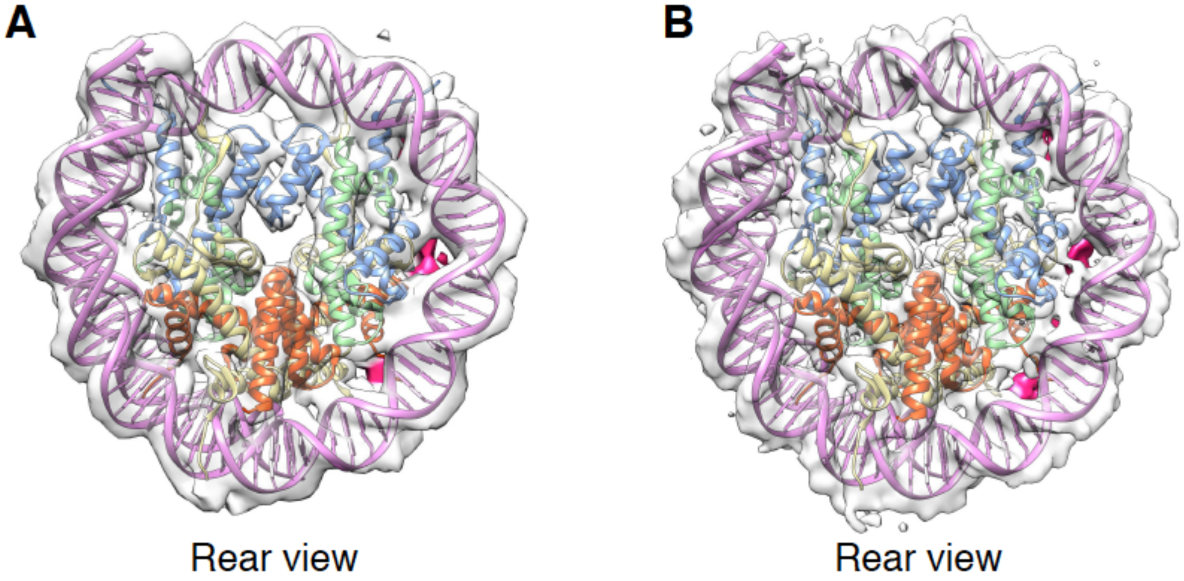

Figure S8

Validation of cryo-EM structures.

(A, B, C) Two different views of the cryo-EM density maps of the 112-bp octasome/Mid-pAID complex (A), the 112-bp octasome/pAID complex (B), and the 112-bp hexasome (C), colored according to the local resolutions. The pAID segments are encircled with a red dotted oval in (A) and (B). The internal histones are encircled with a red dotted oval in (C). The local resolutions of pAID range from 6 to 9 Å for the octasome/Mid-pAID complex (A) and from 5 to 9 Å for the octasome/pAID complex (B), respectively.

(D, E, F) Structural models of histones and the 112-bp DNA in the nucleosome structure (PDB: 2CV5) are fitted into the cryo-EM density maps of the 112-bp octasome/Mid-pAID complex (D), the 112-bp octasome/pAID complex (E), and the 112-bp hexasome (F), colored as in Figure 1B.

(G, H, I) Euler angle distributions of all particles used for 3D reconstruction of the 112-bp octasome/Mid-pAID complex (G), the 112-bp octasome/pAID complex (H), and the 112-bp hexasome (I).

Figure S8

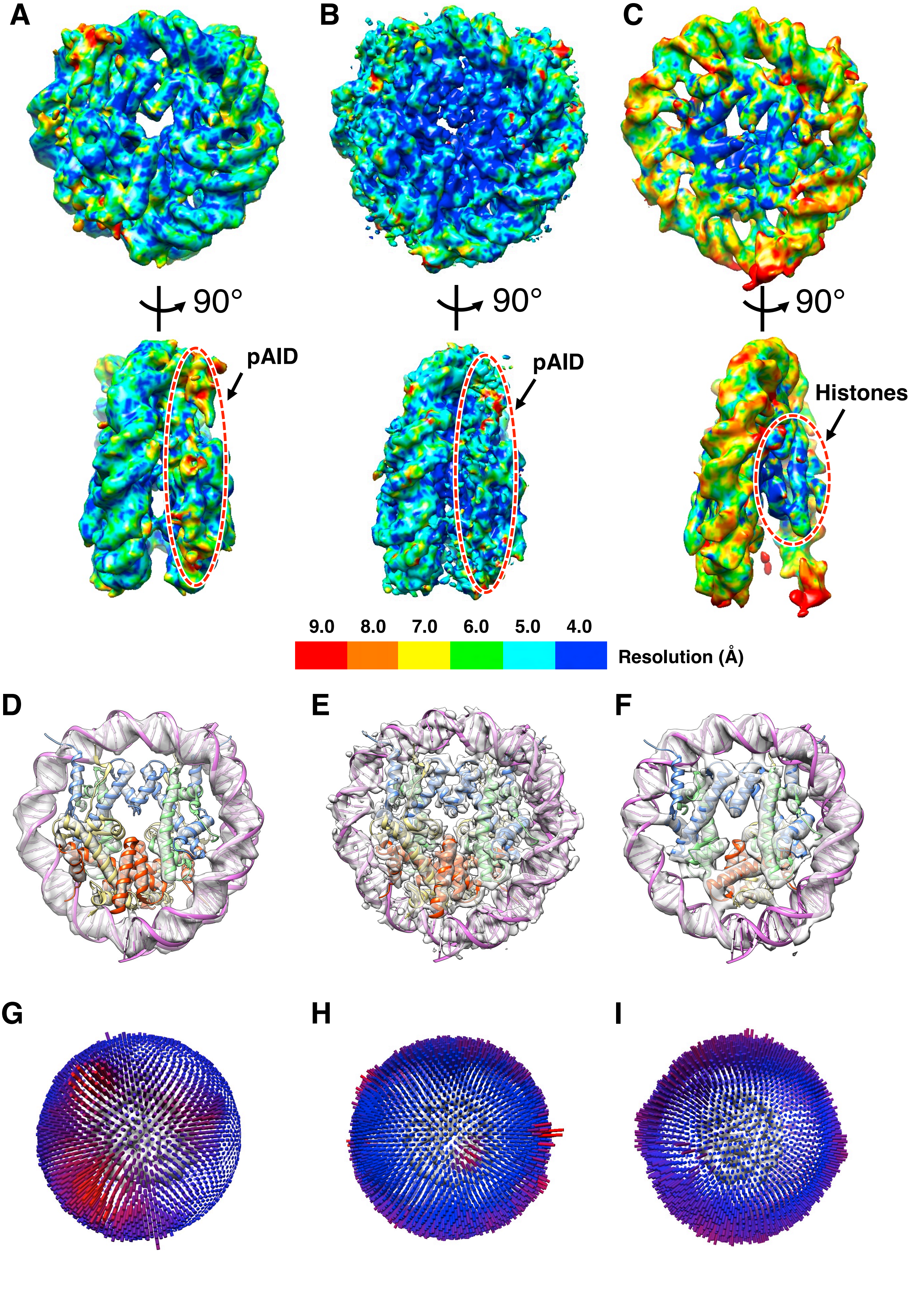
A
B
C
90°
90°
90°
pAID
pAID
Histones
9.0
8.0
7.0
6.0
5.0
4.0
Resolution (Å)
D
E
F
G
H
I

Figure S9

Close-up views of the 112-bp octasome/pAID complex, colored as in Figure 2B.

(A) Close-up views of histone helices. The side chains are depicted. Automated B-factor sharpening[53] in Relion was adopted to calculate this density map (B-factor = -280).

(B, C, D) Close-up views of pAID-histone contacts, showing around the H3 αN helix (B), the H2A L2 loop and the H2B αN helix (C), and the H2A αN helix and the H2A α1 helix (D). pAID appear to be in contact with some side chains in histone proteins (H39, Y41, R42, R49 and K56 of H3, R30 and Y39 of H2B, and R17, R20, R29, R32, R35, K75, and R77 of H2A). These residues are depicted.

Figure S9

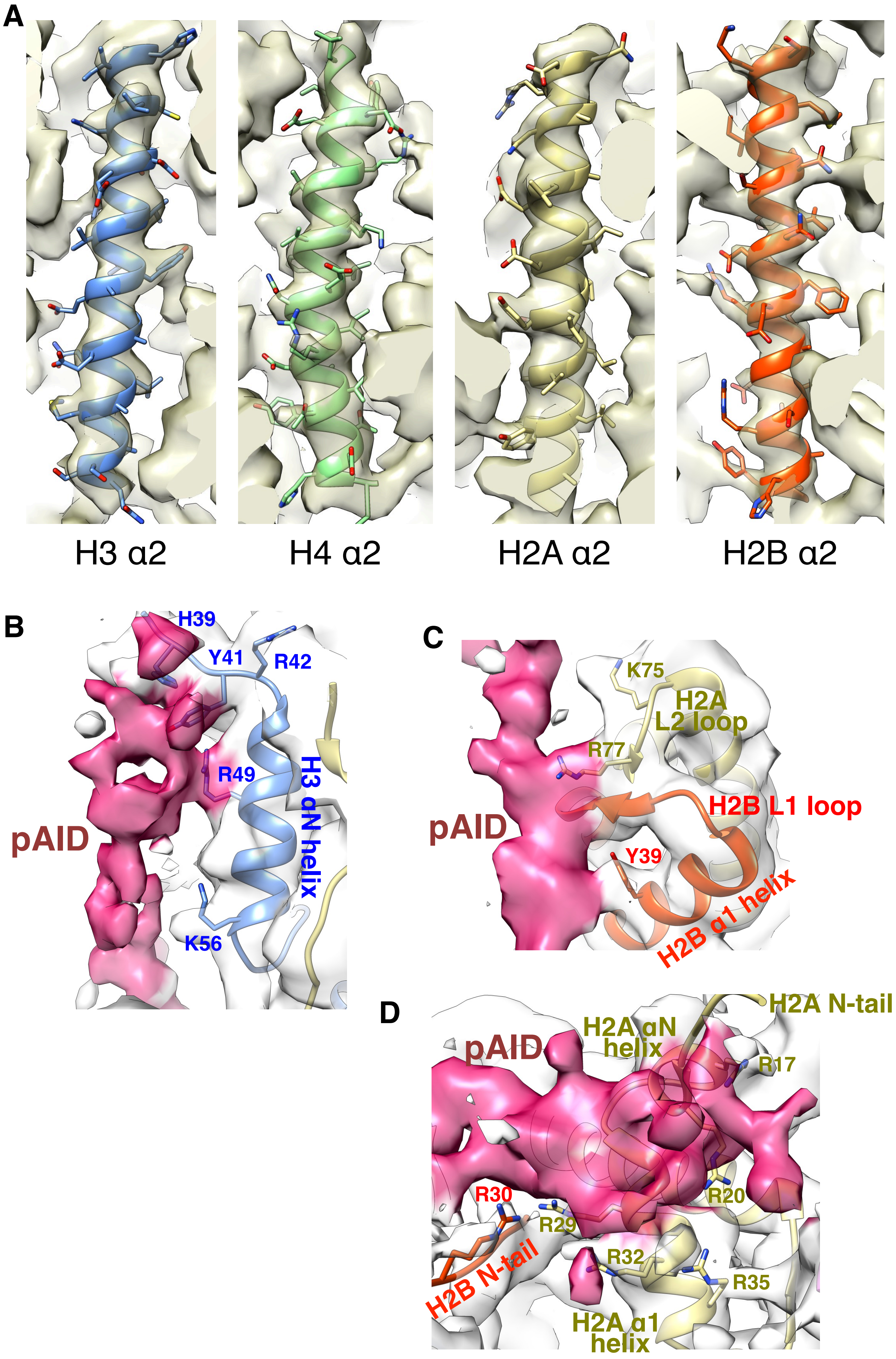
A
H3 α2
H4 α2
H2A α2
H2B α2
B
H39
Y41
R42
R49
H3 αN helix
pAID
K56
C
K75
H2A
L2 loop
R77
H2B L1 loop
pAID
Y39
H2B α1 helix
D
H2A N-tail
H2A αN
helix
pAID
R17
R30
R20
R29
R32
R35
H2B N-tail
H2A α1
helix

Figure S10

Approximately one-half of the human nucleosome structure (PDB ID: 2CV5), colored as in Figure 1B. The missing 43-bp DNA is marked by blue arrows.

Figure S10

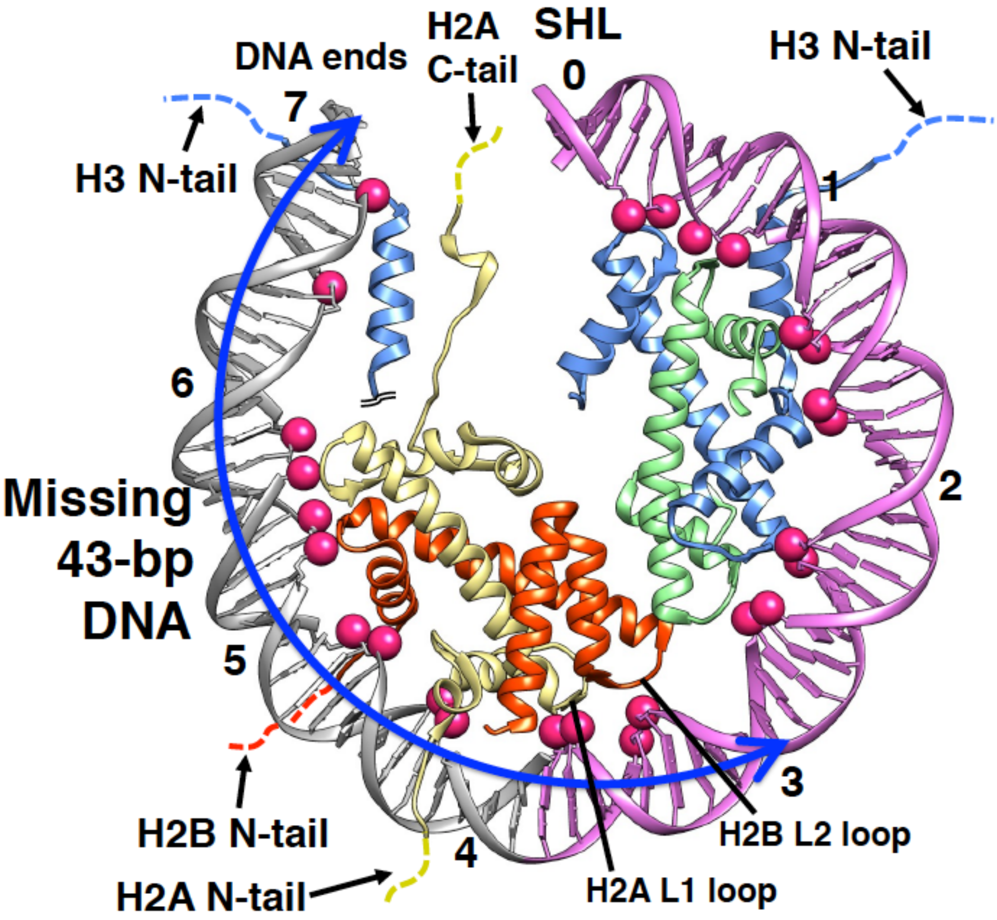

DNA ends
7
H2A
C-tail
SHL
0
H3 N-tail
H3 N-tail
1
6
2
Missing
43-bp
DNA
5
3
H2B N-tail
4
H2B L2 loop
H2A N-tail
H2A L1 loop

Figure S11

Analysis of the complex formation of the FACT proteins with the truncated mutants of histone tails within the 112-bp octasome.

EMSAs show the complexes of the FACT proteins or the 33-bp 601 DNA with the truncated mutants of a histone tail within the 112-bp octasome (H3_ΔN-tail or H2B_ΔN-tail), detected by SYBR Gold nucleic acid gel stain. Experiments were repeated at least twice.

Figure S11

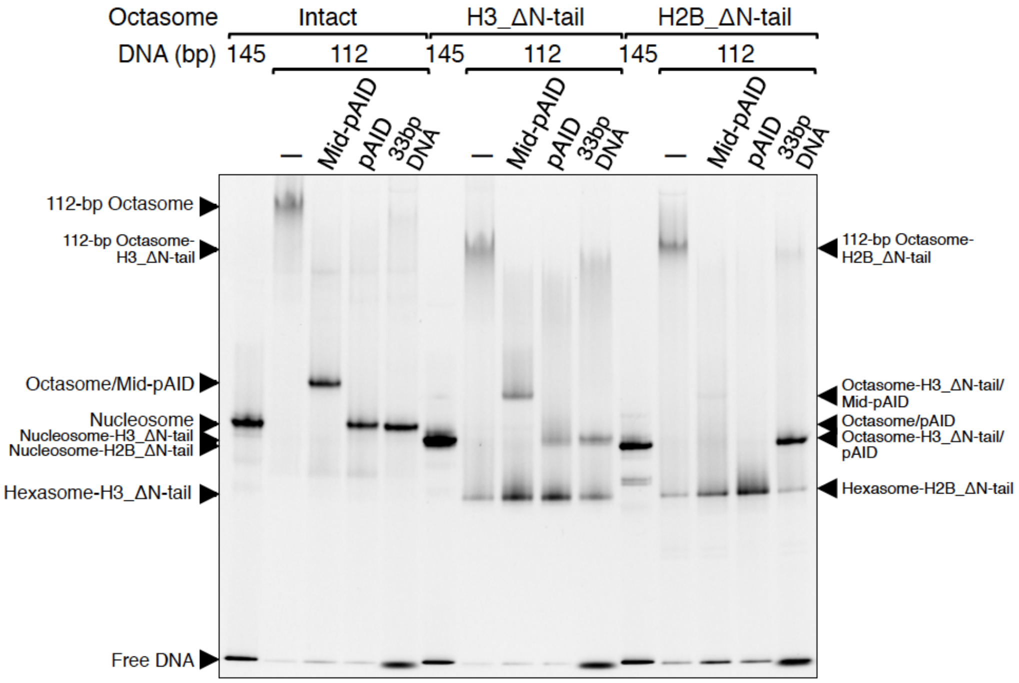

Octasome
Intact
H3_ΔN-tail
H2B_ΔN-tail
DNA (bp)
145
112
145
112
145
112
—
Mid-pAID
pAID
33bp DNA
—
Mid-pAID
pAID
33bp DNA
—
Mid-pAID
pAID
33bp DNA
112-bp Octasome
112-bp Octasome-H3_ΔN-tail
Octasome/Mid-pAID
Nucleosome
Nucleosome-H3_ΔN-tail
Nucleosome-H2B_ΔN-tail
Hexasome-H3_ΔN-tail
Free DNA
112-bp Octasome-H2B_ΔN-tail
Octasome-H3_ΔN-tail/Mid-pAID
Octasome/pAID
Octasome-H3_ΔN-tail/pAID
Hexasome-H2B_ΔN-tail

Figure S12

Comparison of our hexasome map (C) with other hexasome maps (A, B).

These cryo-EM maps [**(A)** class 9 (EMDB code 3925) map at 8.3 Å resolution; **(B)** class 8 (EMDB code 3926) map at 10.5 Å resolution] were determined by Bilokapic et al.[21]. **(C)** Structural models of histones and the 112-bp DNA in the nucleosome structure (PDB: 2CV5) are fitted into our cryo-EM maps at 7.5 Å resolution.

Figure S12

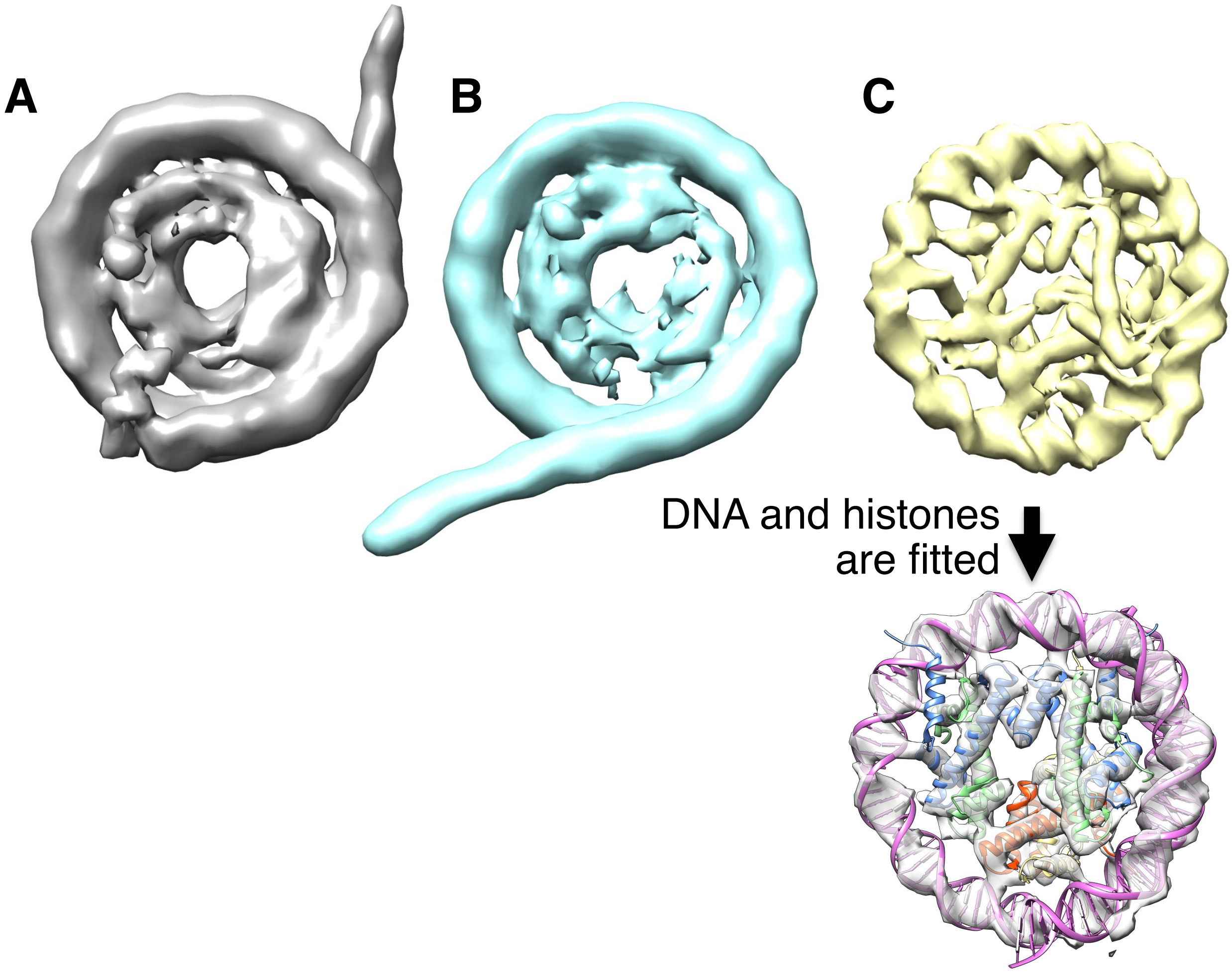

Figure S13

Flow charts of cryo-EM data processing for the 112-bp octaome.

(A, B, C) Representative cryo-electron micrographs of the 112-bp octasome, recorded by a CCD camera, without VPP (A, B) and recorded by a Falcon 3EC direct electron detector using the EC mode, without VPP (C). Images mostly show aggregates in (A), oligomers in (B), and dissociated DNA strands in (B). Red and white dotted circles show representative particles with mono-nucleosome and oligo-nucleosome sizes, respectively.

(D) 2D class averages for particles in cryo-electron micrographs including (B). The details are described in Methods. Red dotted circles show representative particles with the mono-nucleosome size. The others appear to show oligomers of octasomes.

(E) 3D classification and refinement procedures for mono-nucleosome size particles in cryo-electron micrographs including (C). The details are described in Methods. EM structures viewed from front and back sides are shown in their classification scheme.

(F) Comparison between representative 2D class averages (top) and projections from the 3D model (bottom) of the 112-bp octasome/DNA complex in Figure S14A.

(G) Fourier shell correction (FSC) curves for the EM density maps of the 112-bp octasome/DNA complex in Figure S14A. Resolutions are given for the FSC 0.143 criteria.

Figure S13

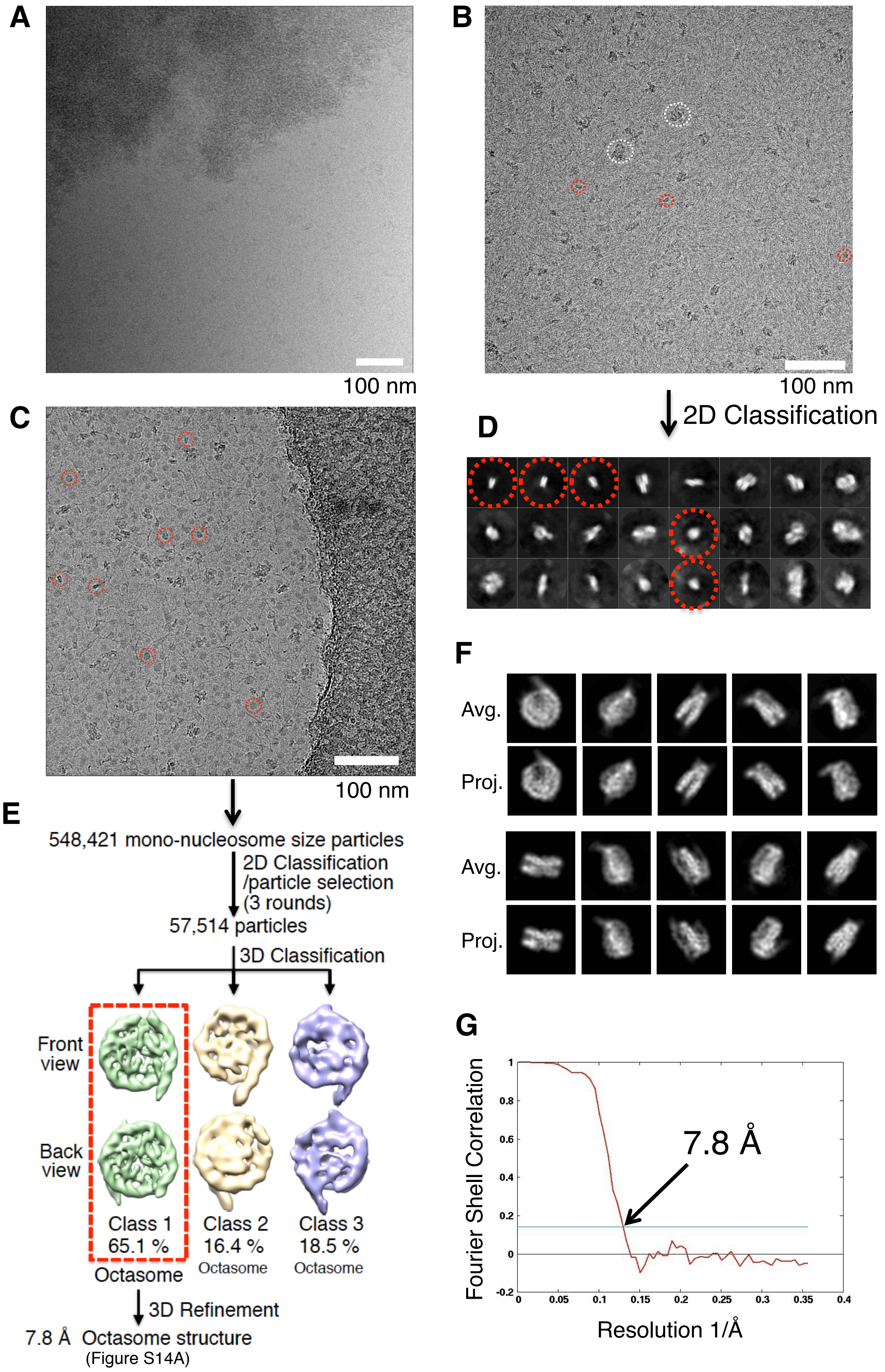
A
100 nm
B
100 nm
2D Classification
C
100 nm
D
E
548,421 mono-nucleosome size particles
2D Classification
/particle selection
(3 rounds)
57,514 particles
3D Classification
Front view
Back view
Class 1
65.1 %
Octasome
Class 2
16.4 %
Octasome
Class 3
18.5 %
Octasome
3D Refinement
7.8 Å Octasome structure
(Figure S14A)
F
Avg.
Proj.
Avg.
Proj.
G
Fourier Shell Correlation
7.8 Å
Resolution 1/Å
1
0.8
0.6
0.4
0.2
0
-0.2
0
0.05
0.1
0.15
0.2
0.25
0.3
0.35
0.4

Figure S14

Cryo-EM structure of the 112-bp octasomes complexed with an extra DNA duplex. The cryo-EM images of the 112-bp octasome mostly showed dissociated DNA strands, in addition to oligomers and aggregates (Figure S13A-D). Nevertheless, some particles with the mono-nucleosome size were ~~partly~~ observed (Figure S13B-D). The reconstructed 3D images of these particles revealed octasome-like structures (Figure S13E). For these structures, the resulting density at an overall resolution of 7.8 Å (Table 1 and Figures S13F,G and S14) fit well to the 112-bp octasome structure and an additional DNA duplex, which binds to the exposed histone surface on the octasome. It is no wonder that the structure shows a DNA gap; we interpreted the data as showing that the complex accommodates part of the free 112-bp DNA duplex, which is derived from the 112-bp octasomes disassembled under the cryo-EM conditions. Such a structure was not found in the presence of the FACT proteins (Figure S6) and in the native MS measurements for the 112-bp octasome (Figure 1C). These results also imply that the cryo-EM procedure, with its tendency to denature the 112-bp octasome, may artificially produce irregular oligomers or aggregates of octasomes.

(A) Cryo-EM structure of the 112-bp octasome/DNA complex. Two different views of the cryo-EM density map are superimposed onto the nucleosome structure (PDB ID: 2CV5, colored as in Figure 1B). The extra DNA duplex is indicated by sky blue. We presume that the complex would accommodate a part of the free 112-bp DNA duplex, generated from the octasome in the specimen preparation. Since the density at both ends of the extra DNA is blurred out by the averaging process (sky blue dotted lines), one end of the extra DNA appears to be detached from the histone core for 3 base pairs (red arrow). Thus, a DNA gap implies that a strong positioning sequence within the 112-bp DNA happened to bind to the histone surface.

(B) Two different views of the cryo-EM density maps of the 112-bp octasome/DNA complex, colored according to the local resolutions.

(C) Euler angle distributions of all particles used for 3D reconstruction of the 112-bp octasome/DNA complex.

Figure S14

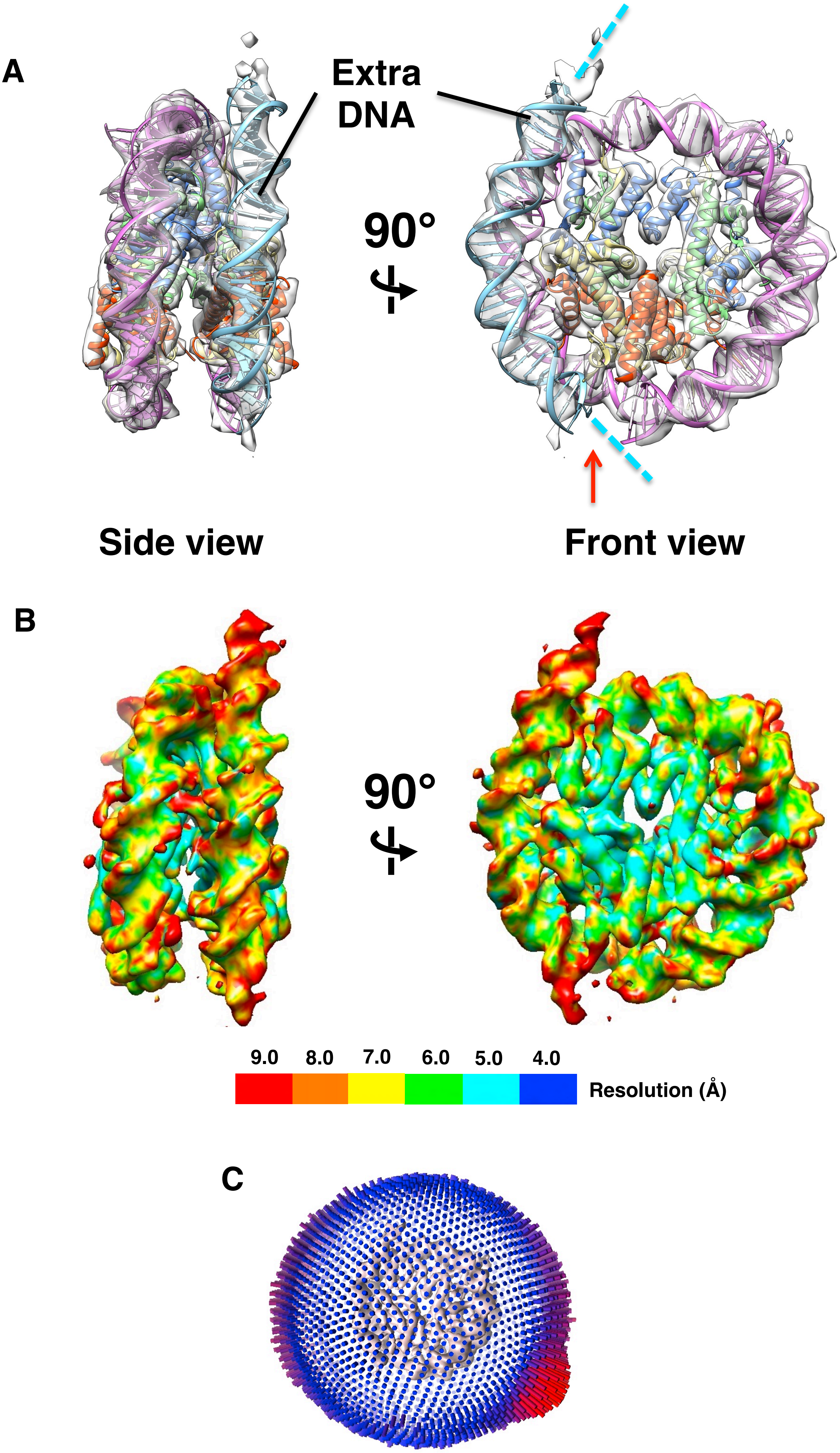
A
Extra
DNA
90°
Side view
Front view
B
90°
9.0
8.0
7.0
6.0
5.0
4.0
Resolution (Å)
C

Figure S15

Ion mobility contour plot of the 112-bp octasome reconstituted with pAID.

In the Ion mobility contour plot, the m/z values (x-axis, Figure 1G) were plotted vs. the arrival times (y-axis). Relative abundances of the ions are indicated by a color gradation. The Ion mobility contour plot of the 112-bp octasome reconstituted with pAID showed a single component, corresponding to the 112-bp octasome complexed with pAID.

Figure S15

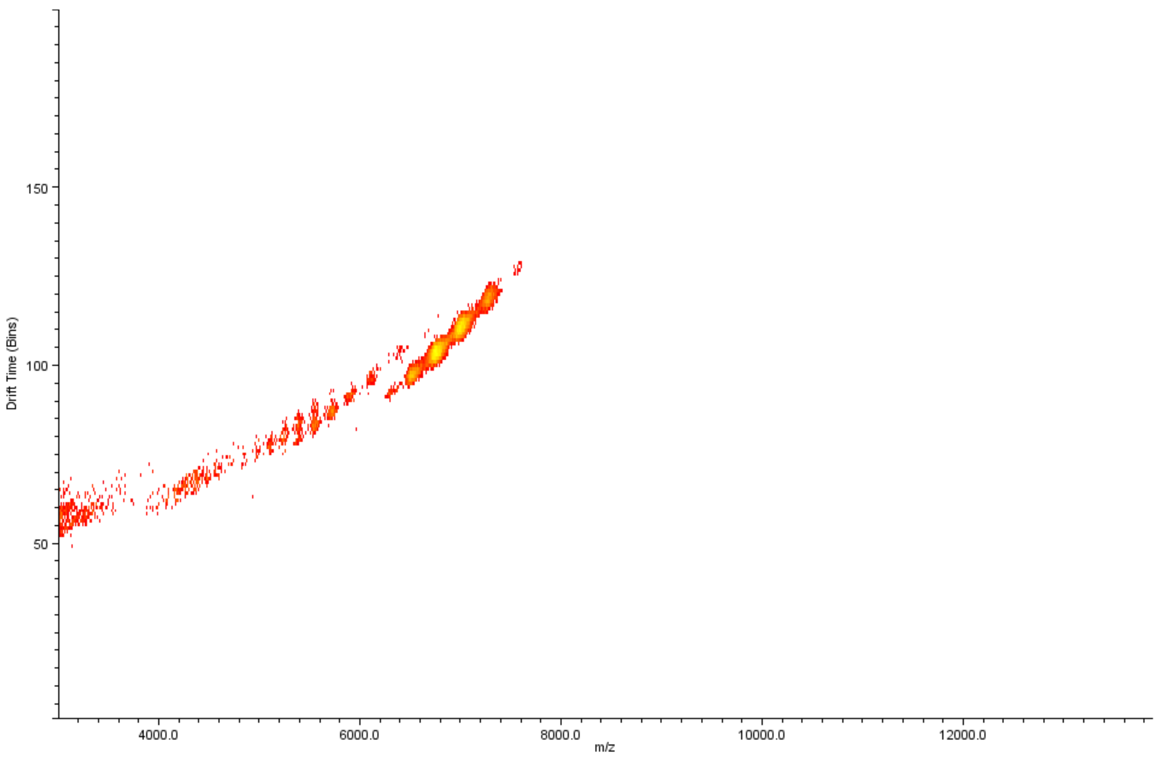

150
100
50
Drift Time (Bins)
4000.0
6000.0
8000.0
10000.0
12000.0
m/z

Figure S16

Representation of the docking model of nucleosome with Mid.

The model was constructed by making the best superposition of the $(H3\text{-}H4)_2$ structure between Mid-pAID/$(H3\text{-}H4)_2$ (PDB code 4Z2M) and nucleosome (PDB code 2CV5), as described previously[20]. Nucleosomes are represented in ribbon models of H3 (light blue), H4 (green), and DNA (gray). The two copies of H2A-H2B and the DNA bases are removed for clarity. Mid is shown as an electrostatic potential surface, colored between -7.0 kT/e (red) and 7.0 kT/e (blue). In the front view, the electrostatic potential surface is represented with 70% transparency for clarity. Red circles show the locations of steric hindrance between the nucleosomal DNA and the H3-H4 binding loop of Mid. The indicated residues (R674 and R675) are mapped on a broad basic surface of Mid in contact with the nucleosomal DNA around the central region.

Figure S16

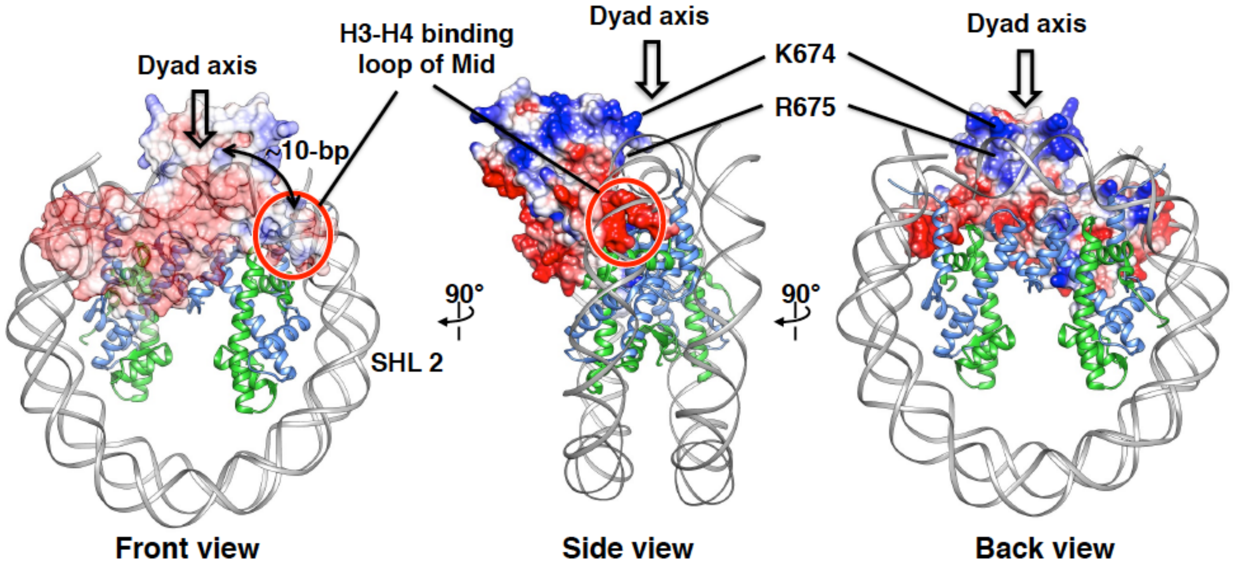

H3-H4 binding
loop of Mid
Dyad axis
~10-bp
SHL 2
90°
Dyad axis
K674
R675
90°
Dyad axis
Front view
Side view
Back view
